# Flow and fracture behavior of aluminum alloy 6082-T6 at different tensile strain rates and triaxialities

**DOI:** 10.1371/journal.pone.0181983

**Published:** 2017-07-31

**Authors:** Xuanzhen Chen, Yong Peng, Shan Peng, Song Yao, Chao Chen, Ping Xu

**Affiliations:** 1 Key Laboratory of Traffic Safety on Track of Ministry of Education, School of Traffic & Transportation Engineering, Central South University, Changsha, China; 2 State Key Laboratory of High Performance Complex Manufacturing, Central South University, Changsha, China; 3 Hunan Industry Polytechnic, Changsha, China; Beihang University, CHINA

## Abstract

This study aims to investigate the flow and fracture behavior of aluminum alloy 6082-T6 (AA6082-T6) at different strain rates and triaxialities. Two groups of Charpy impact tests were carried out to further investigate its dynamic impact fracture property. A series of tensile tests and numerical simulations based on finite element analysis (FEA) were performed. Experimental data on smooth specimens under various strain rates ranging from 0.0001~3400 s^-1^ shows that AA6082-T6 is rather insensitive to strain rates in general. However, clear rate sensitivity was observed in the range of 0.001~1 s^-1^ while such a characteristic is counteracted by the adiabatic heating of specimens under high strain rates. A Johnson-Cook constitutive model was proposed based on tensile tests at different strain rates. In this study, the average stress triaxiality and equivalent plastic strain at facture obtained from numerical simulations were used for the calibration of J-C fracture model. Both of the J-C constitutive model and fracture model were employed in numerical simulations and the results was compared with experimental results. The calibrated J-C fracture model exhibits higher accuracy than the J-C fracture model obtained by the common method in predicting the fracture behavior of AA6082-T6. Finally, the Scanning Electron Microscope (SEM) of fractured specimens with different initial stress triaxialities were analyzed. The magnified fractographs indicate that high initial stress triaxiality likely results in dimple fracture.

## Introduction

Aluminum alloys, with low density, high strength to weight ratio, good ductility and excellent corrosion resistance, are widely used in aviation, aerospace, automotive, machinery manufacturing etc. 6082-T6 aluminum alloy, as an Al-Mg-Si alloy, is increasingly applied to manufacture high-speed trains due to its sufficient plasticity for extrusion, relatively high strength, excellent weldability, formability and machinability. It is well known that aluminum alloys have some mechanical properties like the dependence of mechanical properties on the strain rate, stress state and temperature. Spigarelli et al. [1] analyzed the peak stress dependence on strain rate and temperature based on a series of torsion tests in a wide range of temperatures and strain rates. With regard to the 6082 aluminum mechanical properties, several fruitful studies were conducted, such as Tranə et al. [2], who have evaluated the drawing efficiency of 6082 0 temper aluminum alloy for cartridge tubes. They applied the Piecewise-Linear-Plasticity constitutive model and the Cockcroft-Latham fracture criterion to the simulations. Two important conclusions were drawn in their work that the stress triaxiality ratio dominated the material failure and the constitutive model for 6082 0 temper aluminum alloy was an exponential law. For 6082 aluminum alloy in T6 temper (solution heat treatment and artificial aging), little rate sensitivity and moderated anisotropy was found by Chen et al. [3].

Considerable quantity of classic researches [4–6] have been done to study the relation between mechanical properties and the strain rate. Comparing with other constitutive relations, the model proposed by Johnson and Cook [6], which describes the material stress response at large strain, various strain rates and elevated temperatures, is widely used in impact problems. Also, they provided material parameters of 12 different materials, Hu et al. [7] determined those for AerMet 100 steel, Feng et al. [8] for AZ31B magnesium alloy, Trajkovski et al. [9] for armor steel PROTAC 500. Singh et al. [10] derived the J-C parameters based on the dynamic increase factors of strengths for CP800 steel. Some authors, including Børvik et al. [11], Hou and Wang [12], and Li et al. [13] proposed modified J-C constitutive models to predict material mechanical response.

Fracture ductility, as the ability of a material to bear plastic deformation without failure, is of great significance for the application of a material. Studies of effect of stress state on fracture ductility may be dated back to the research of Ludwik and Scheu [14]. They hold the view that the strength-strain curve could be given by testing tensile specimens with circumferential notches of different depths. The stress triaxiality is defined as the ratio of the hydrostatic stress and the equivalent stress to depict the stress state of materials. Tests carried out by Bridgman [15] showed that a reduced stress triaxiality could increase the fracture strain greatly. Similar conclusion was drawn by Hancock and Mackenzie [16] through a series of tensile tests on pre-notched round steel specimens.

Several failure criterions have been used in commercial FEA codes by now such as LS-Dyna, Abaqus, and Ansys. The Johnson-Cook failure criterion [17] has been widely used to study the dependence of fracture strain on the strain rate, stress triaxiality, and temperature through both experiments and numerical simulations. Considering the anisotropy of the material, Johnson-Cook failure criterions of AA5083-H116 were proposed by Clausen et al. [18] along different directions of the rolled material, and the 0° direction specimens exhibit an opposite fracture strain tendency for various stress triaxialities with others. Erice et al. [19] calibrated the J-C fracture criterion of FV535 steel by averaging Bridgman’s analysis and the numerical simulation result. Knowing that only high stress triaxiality is considered by the J-C failure criterion, Bao and Wierzbicki [20] studied the fracture ductility in the entire range of stress triaxialities by carrying out a series of upsetting, shear, and tension on 2024-T351 aluminum alloy, and suggested a segmented failure criterion. However, those few published studies of the fracture criterion coupled with dynamic constitutive mechanical behavior cannot match the increasingly engineering application of AA6082-T6. The objective of this paper is to bridge the gap.

In this paper, quasi-static and dynamic tensile tests for AA6082-T6 were carried out. A constitutive model was suggested to describe the dynamic mechanical behavior. Coupled with the constitutive model, a combined experimental and simulation method was used to calibrate the J-C fracture model. Further, the tensile tests were simulated to validate the constitutive model and fracture model. Magnified fractographs of smooth and notched specimens were recorded by SEM. In the end, this paper discusses the mechanical properties of the investigated material under various strain rates and stress triaxialities.

## Experiment and method

### Experimental procedure and materials

#### Quasi-static and low strain rate tests

Experiments conducted in this study consist of axial tensile tests and notched specimen tensile tests. The former groups were set to study the influence of strain rate on mechanical behavior of aluminum alloy AA6082-T6, and the last one is for the influence of stress triaxiality on ductility. Three repeated experiments in each case were conducted to ensure the test reliability.

**Fig 1:**
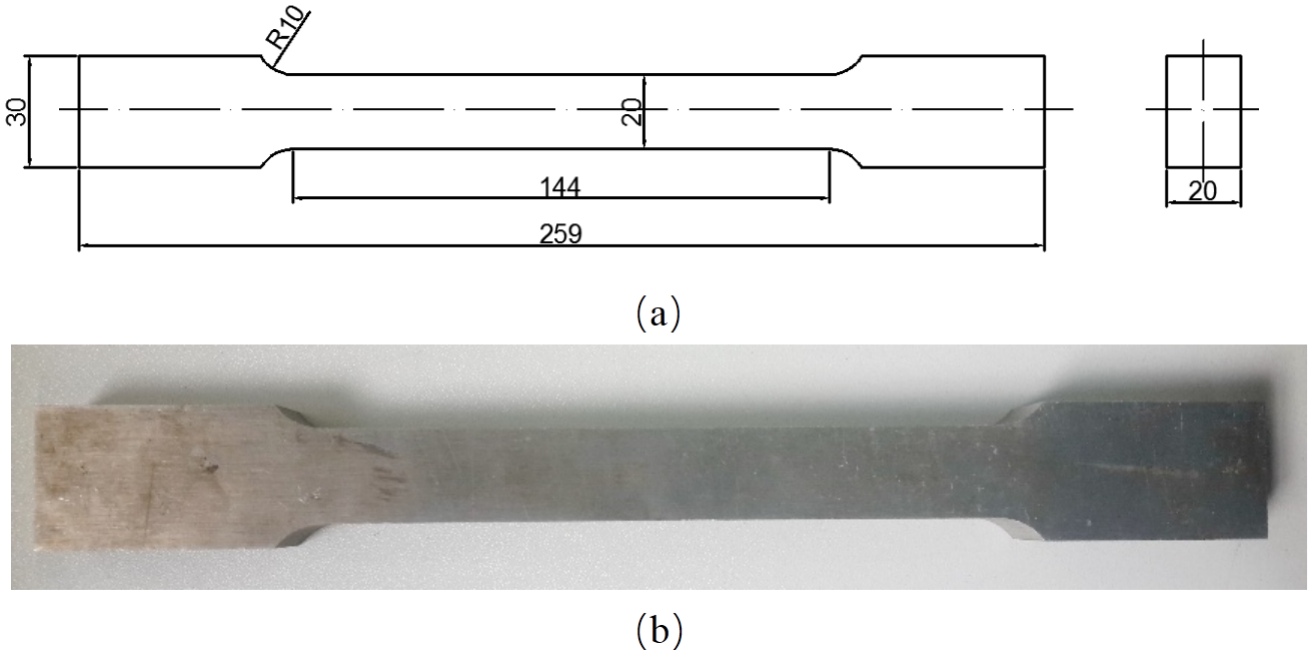
Flat specimens (a) geometry (b) specimens (unit: mm).

The chemical compositions (in wt. %) of aluminum alloy AA6082-T6 used in this study are listed in Table 1. The original material was rolled sheet, and all the specimens for the tensile experiment were cut with the length direction of the specimens coincident with the rolling direction of the sheet. For quasi-static (0.0001 s^-1^) and low strain rate (0.001~1 s^-1^) tension tests, the rectangular cross section flat specimens were used with total length of 259 mm, minimum width of 20 mm and thickness of 20 mm, as shown in Fig 1.

**Table 1:** The chemical compositions of aluminum alloy 6082-T6.

Chemical element	Si	Fe	Cu	Mn	Mg	Cr	Zn	Ti
wt.%	0.8	0.5	0.1	0.7	0.9	0.25	0.2	0.1

**Fig 2:**
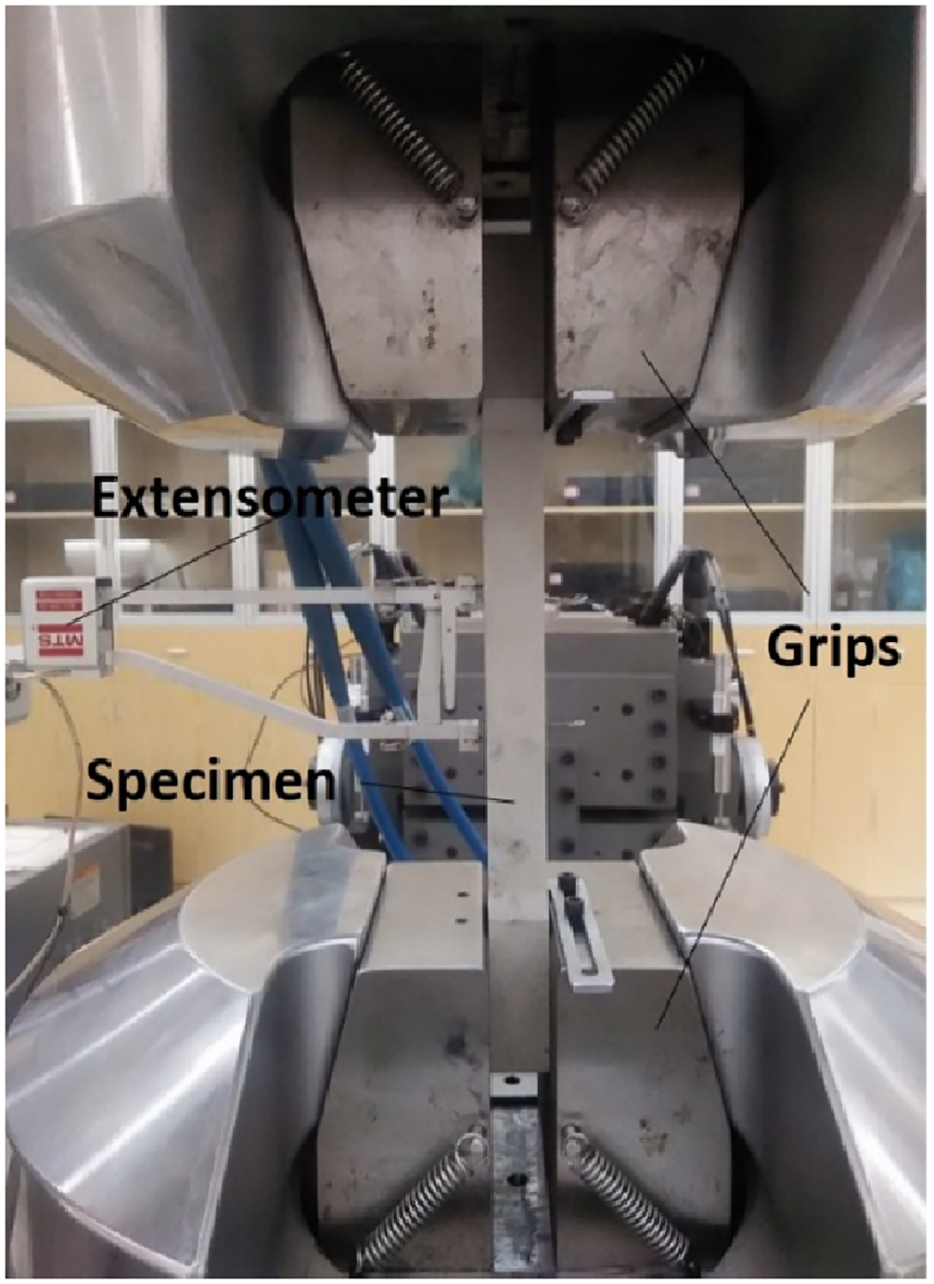
Test photo of MTS electronic universal testing machine.

The tensile tests were performed by MTS electronic universal testing machine (shown in Fig 2.) at room temperature 20°C except high rate (100~10^4^ s^-1^) tensile tests. The specimens were clamped with a wedge-shaped clamp in tension experiments, as shown in Fig 2. The displacement was measured by an extensometer with the resolution of 0.5 μm.

#### High strain rate tensile tests

**Fig 3:**
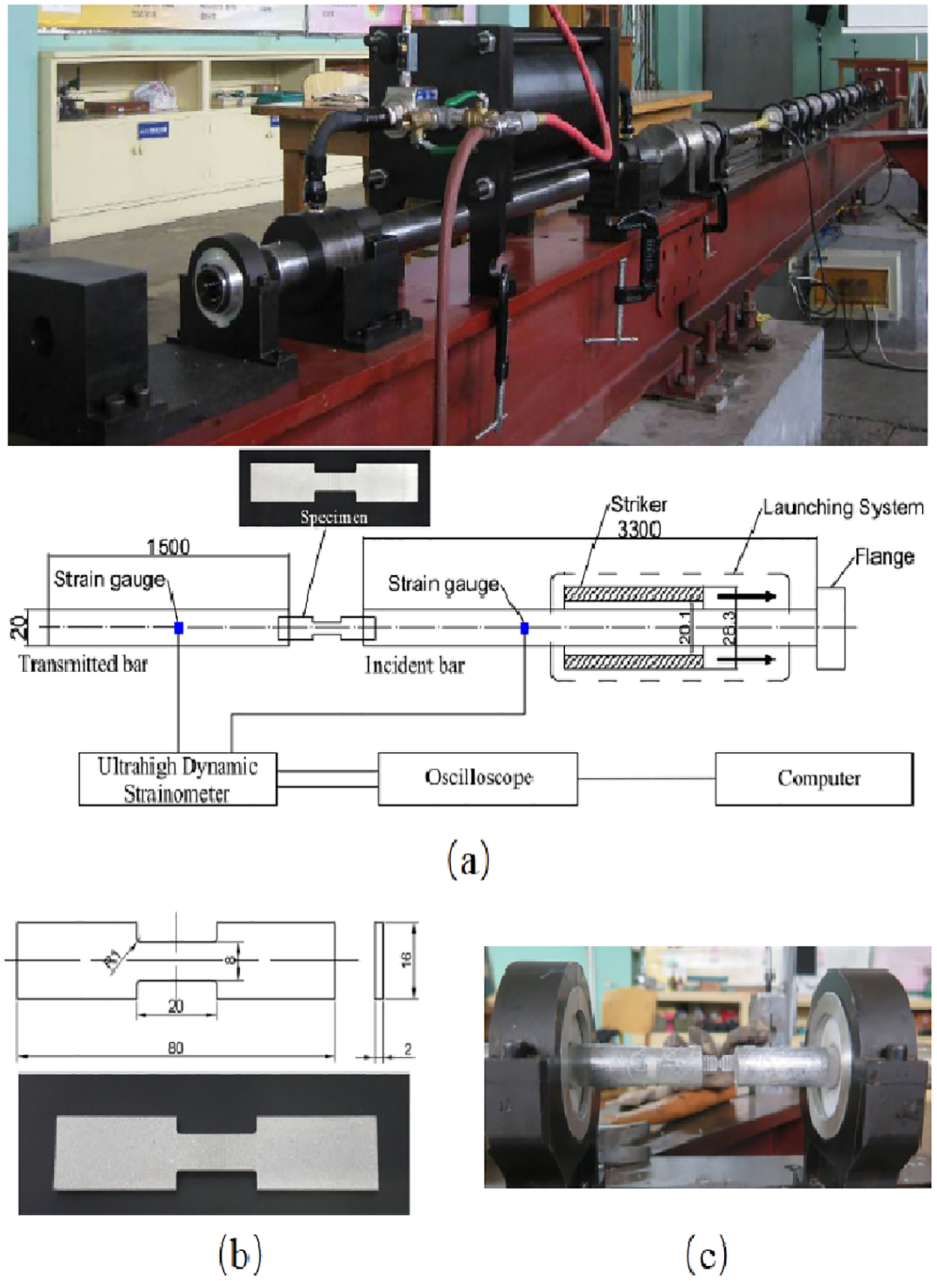
(a) Split Hopkinson pressure bar (SHTB) testing apparatus; (b) The geometry of the SHTB-specimen; (c) A notched specimen sandwiched between the incident bar and the transmission bar.

**Fig 4:**
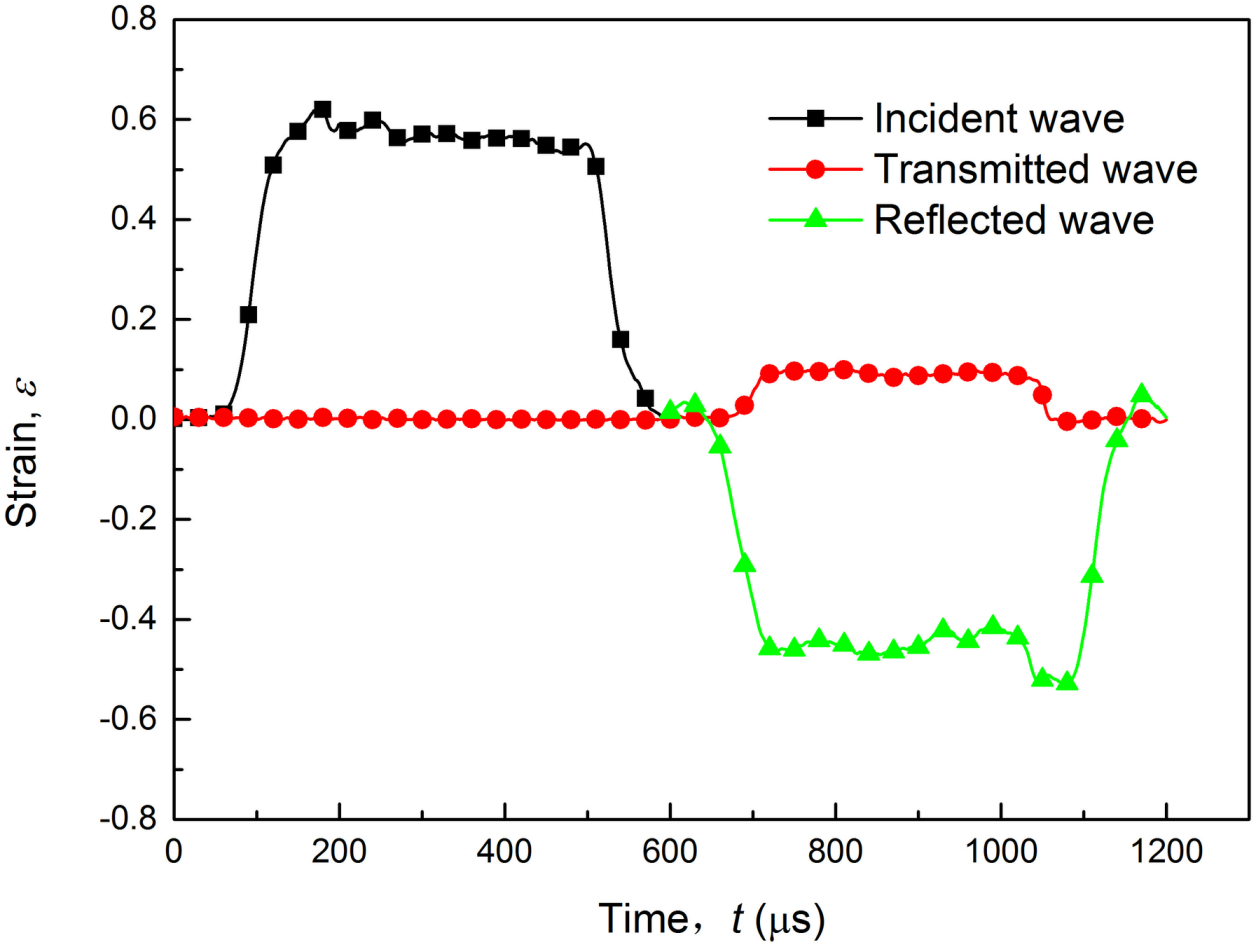
Typical experimental strain waves of SHTB testing at strain rate of 800 s^-1^.

For high rate tensile tests, a split Hopkinson tension bar (SHTB) testing apparatus was used to characterize the dynamic properties of the investigated material. The SHTB (shown in Fig 3(a)) device mainly consists of a striker, an incident bar, a transmitted bar, strain gauge, digital storage oscilloscope and an ultrahigh dynamic strainometer. Fig 3(b) is the geometry of the flat SHTB-specimen. Fig 3(c) shows a specimen sandwiched between the incident bar and transmission bar. The pressure bars are made of LY12 cryogenic aluminum alloy. The incident bar is 3300 mm in length and the transmitted bar is 1500 mm. The diameter is 20 mm for both bars. The tubular striker is made of the same material as the pressure bars. When the striker is propelled by the gas gun impacting the incident bar, an elastic wave is formed and travels through the incident bar. Once the wave reaches the specimen, due to the interaction with the specimen, part of the wave is reflected back into the incident bar and the remaining part, the transmitted wave, passes through the transmission bar. In the process of wave propagation, the specimen is under high rate tension, and the strain histories of those waves (the incident, reflected and transmitted wave) are recorded by the strain gauges mounted on both bars and collected by Tektronix 4104 digital storage oscilloscope. Fig 4 shows the strain waves at a strain rate of 800 s^-1^.

In the high strain rate tensile tests, the strain rate was controlled by changing the length of the striker and the air pressure of the gas gun. The striker length varies from 300 mm to 1050 mm. The tests were conducted at three different strain rates, i.e., 800 s^-1^, 2700 s^-1^ and 3400 s^-1^. Three repeated tests under the same loading condition were carried out to ensure the reliability of the results.

According to the principles of one-dimensional elastic-wave propagation in slender bars, the engineering stress, the engineering strain and the strain rate can be calculated from the reflected and transmitted wave amplitude via the following equations [10]

$$\sigma_{e}\left(t\right)=E\frac{A_{0}}{A_{S}}\varepsilon_{tw}\left(t\right)$$ (1a)

$$\varepsilon_{e}\left(t\right)=-\frac{2C_{0}}{L}\int_{0}^{t}\varepsilon_{tw}\left(t\right)dt$$ (1b)

$$\dot{\varepsilon }\left(t\right)=-\frac{2C_{0}}{L}\varepsilon_{rw}\left(t\right)$$ (1c)

where *σ*_*e*_(*t*), *ε*_*e*_(*t*) and $\dot{\varepsilon }\left(t\right)$ are the engineering stress, the engineering strain and the strain rate, respectively. *ε*_*tw*_(*t*) and *ε*_*rw*_(*t*) refer to the amplitude of the transmitted wave and the reflected wave. *E*, *C*_0_ and *A*_0_ are Young’s modulus, the stress wave speed and cross section area of the incident bar, respectively. *A*_*S*_ and *L* represent cross section area and length of the specimen, respectively.

The true stress and true strain (respectively denoted as *σ*_*t*_ and *ε*_*t*_) are given by the following equations

$$\sigma_{t}\left(t\right)=\sigma_{e}\left(t\right)\left(1+\varepsilon_{e}\left(t\right)\right)$$ (2a)

$$\varepsilon_{t}\left(t\right)=\text{ln}\left(1+\varepsilon_{e}\left(t\right)\right)$$ (2b)

### Notched specimen tensile tests

Studies [14–20] have been performed in the past that proved fracture ductility depends markedly on the triaxiality of the stress state. The triaxiality is usually represented by the dimensionless stress triaxiality ratio *η*, which is defined as

$$\eta =\frac{\sigma_{m}}{\sigma_{eq}}=\frac{\left(\sigma_{1}+\sigma_{2}+\sigma_{3}\right)/3}{\sqrt{\frac{{\left(\sigma_{1}-\sigma_{2}\right)}^{2}+{\left(\sigma_{2}-\sigma_{3}\right)}^{2}+{\left(\sigma_{3}-\sigma_{1}\right)}^{2}}{2}}}$$ (3)

where *σ*_*m*_ is the hydrostatic stress, *σ*_*eq*_ is the von Mises equivalent stress, and *σ*_1_, *σ*_2_, *σ*_3_ represent three principal stresses. For an uniaxial stress state where *σ*_1_ is the only non-zero component, stress triaxiality *η* = 1/3.

**Fig 5:**
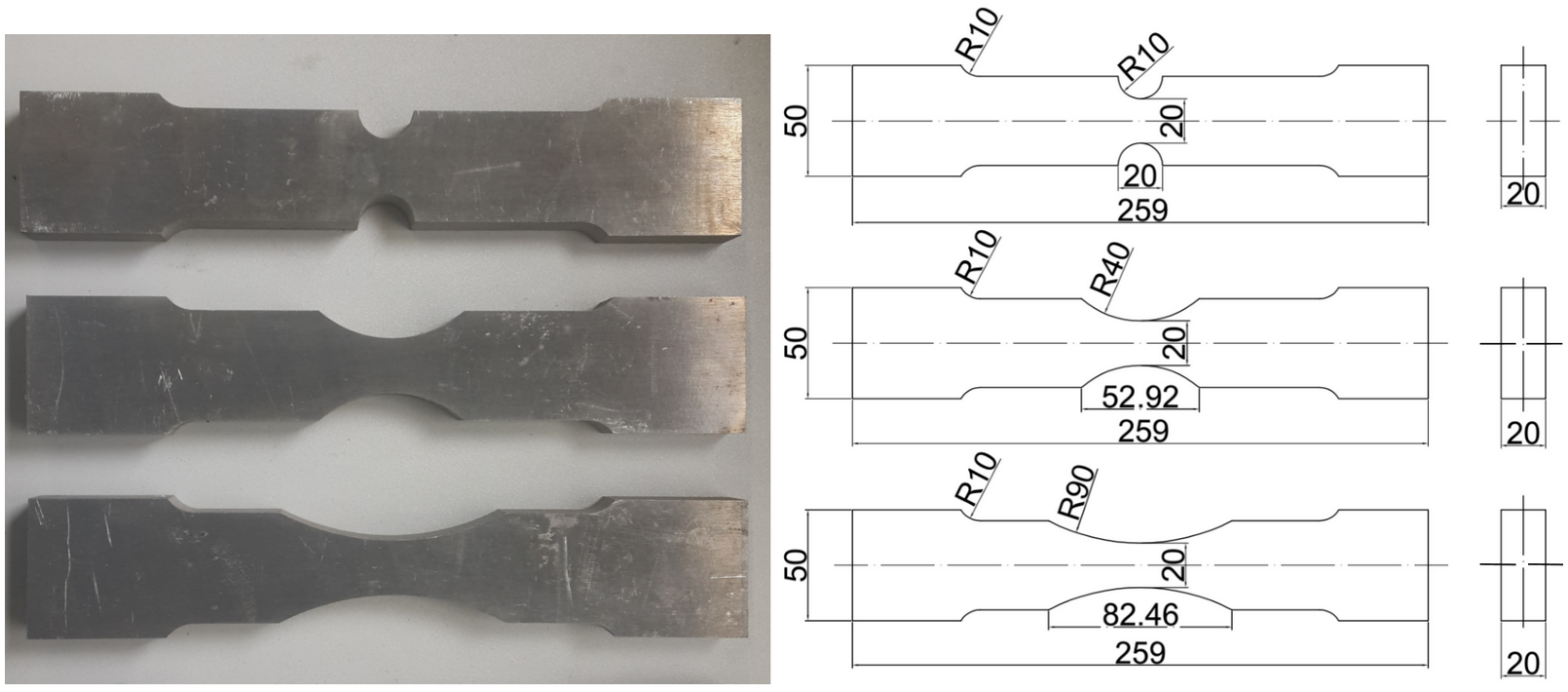
Geometry and dimensions of pre-notched specimens (unit: mm).

Hancock and Mackenzie [16] carried out a series of tensile tests on round pre-notched steel specimens. The initial stress triaxiality was calculated according to Bridgman’s [15] analysis in their study.

$$\eta =\frac{1}{3}+ln\left(\frac{r}{2R}+1\right)$$ (4)

where, *r* is the radius of the minimum cross-section and *R* is the radius of the circumferential notch. As (Eq 4) shows, for round pre-notched specimens the value of stress triaxiality increases when the radius of the notch decreases. To investigate the relation between fracture strain and stress triaxiality of AA6082-T6, three types of flat specimens with different notch size (notch radius 10mm, 40mm, 90mm respectively) were machined (Fig 5) for tensile tests. Because of the different cross-section geometry of round and flat specimen, Bridgman’s analysis is not suitable here. But the magnitude of the notch radius has the same influence on the stress triaxiality. That is, the specimen with the smaller notch radius exhibits the higher value of stress triaxiality. For each type of pre-notched specimens, tensile tests under quasi-static tensile loading were performed. The experimental conditions were exactly the same as those for the previously stated tensile tests on smooth specimens.

### Material model

#### Johnson-Cook constitutive relation

The Johnson-Cook constitutive model, which is an empirical viscoplastic constitutive model, can well depict the work hardening, strain rate hardening and the thermal softening effect of metal materials. The J-C constitutive relation is shown as follows,

$$\sigma =\left(A+B\varepsilon^{n}\right)\left(1+C\text{ ln }{\dot{\varepsilon }}^{*}\right)\left[1-{\left(T^{*}\right)}^{m}\right]$$ (5)

where *σ* is the stress; *ε* is the equivalent plastic strain; ${\dot{\varepsilon }}^{*}$ is the dimensionless plastic strain rate given by the equation of ${\dot{\varepsilon }}^{*}\;=\;\dot{\varepsilon }/{\dot{\varepsilon }}_{0}$ in which ${\dot{\varepsilon }}_{0}$ is the reference strain rate (0.0001 s^-1^); T* is the dimensionless temperature expressed as T* = (*T* − *T*_0_)/(*T*_*m*_ − *T*_0_), in which *T*, *T*_0_, *T*_*m*_ are environment, reference and material melt temperature respectively; *A* is the yield stress; *B* is the strain hardening coefficient; *n* is the strain hardening exponent; *C* is the rate sensitivity coefficient. During high strain rate loading specimens are reasonable to be assumed in adiabatic conditions. In this case the rate of temperature change is calculated through

$$\dot{T}=\chi \frac{\sigma \dot{\varepsilon }}{\rho C_{p}}$$ (6)

where *ρ* and *C*_*p*_ and are the density and heat capacity of material. *χ* is the conversion factor of work into heat. The temperature rise can be estimated using the following equation

$$\int_{T_{0}}^{T}\rho C_{p}dT=\;\chi \int_{0}^{\varepsilon_{e}}\sigma d\varepsilon$$ (7)

where *ε*_*e*_ is the strain corresponding to the maximum stress. *ρ* and *C*_*p*_ are assumed as constants, while *χ* is taken as 0.9 for metals [11]. The equation can be rewritten as

$$\text{T}=T_{0}+\Delta T=T_{0}+\int_{T_{0}}^{T}dT=T_{0}+\frac{0.9}{\rho C_{p}}\int_{0}^{\varepsilon_{e}}\sigma d\varepsilon$$ (8)

In this study all experiments were conducted at room temperature. For tensile tests at quasi-static and low strain rate, the thermal softening effect is neglected for simplicity. Therefore, the constitutive relation can be simplified as

$$\sigma =\left(A+B\varepsilon^{n}\right)\left(1+C\text{ ln }{\dot{\varepsilon }}^{*}\right)$$ (9)

Here, the quasi-static strain rate is taken as 0.0001 s^-1^. For quasi-static experiments, the value of ${\dot{\varepsilon }}^{*}$ can naturally be taken as 1. Therefore, the constitutive model can be further simplified as

$$\sigma =A+B\varepsilon^{n}$$ (10)

where *A* is the true yield stress of the material at a strain rate of 0.0001 s^-1^ which is equal to the stress corresponding to 0.2% offset strain.

By placing constant *A* on the left side of (Eq 10), the following equation can be obtained

$$\sigma -A=B\varepsilon^{n}$$ (11)

(Eq 11) can be used to fit the quasi-static experimental data in plastic deformation stage using the least square method. The values for each *B* and *n* can be directly obtained as parameters *a* and *b* respectively. At room temperature, the value of *C* can be obtained by fitting the strain-stress data of different strain rate experiments. The constitutive model can be rewritten as

$$\sigma_{i}=\sigma_{0}\left(1+C\text{ln}\frac{\dot{\varepsilon }}{{\dot{\varepsilon }}_{0}}\right)$$ (12)

where *σ*_0_ is the yield stress under strain rate 0.0001 s^-1^; *σ*_*i*_ is the yield stress under different strain rates; ${\dot{\varepsilon }}_{0}$ is the reference strain rate (taken 0.0001 s^-1^ as mentioned earlier); $\dot{\varepsilon }$ represents strain rate. (Eq 12) can be converted to the following equation

$$\frac{\sigma_{i}}{\sigma_{0}}-1=C\text{ln}\frac{\dot{\varepsilon }}{{\dot{\varepsilon }}_{0}}$$ (13)

With this method, the constitutive relation of aluminum alloy 6082-T6 without thermal softening effect can be established effectively.

#### Johnson-Cook fracture model

Johnson and Cook proposed a fracture model in which the equivalent plastic fracture strain ε_*f*_ is dependent on strain rate and temperature in addition to stress triaxiality. The general expression for the equivalent plastic strain at fracture given by J-C failure model is

$$\varepsilon_{f}=\left[D_{1}+D_{2}\text{exp}\left(D_{3}\eta \right)\right]\left[1+D_{4}\text{ ln }{\dot{\varepsilon }}^{*}\right]\left[1+D_{5}T^{*}\right]$$ (14)

The constants *D*_1_, *D*_2_, *D*_3_ are determined from the quasi-static tests on smooth and notched axis symmetric specimens and *D*_4_ from various strain rate tests of smooth specimens. In the present study, all tests were conducted at room temperature, temperature influence on fracture strain is thus neglected. The J-C fracture model expression without temperature effect then is simplified as (Eq 15) where only four parameters need to be determined.

$$\varepsilon_{f}=\left[D_{1}+D_{2}\text{exp}\left(D_{3}\eta \right)\right][1+D_{4}\text{ ln }{\dot{\varepsilon }}^{*}]$$ (15)

#### Calibration of the Johnson-Cook fracture model

During tensile tests, the strain field is not homogeneously distributed throughout the whole specimen and the maximum strain occurs on the fracture surface. Constant volume of the specimen and homogenous strain over the fracture surface are assumed and then the fracture strain [19] could be obtained through

$$\varepsilon_{eq}^{f}=\text{ln}\left(\frac{A_{0}}{A_{f}}\right)=\text{ln}\left(\frac{a_{0}b_{0}}{a_{f}b_{f}}\right)$$ (16)

where *A* represent minimum sectional area on the specimen. *a* and *b* are the width and the height of specimens at minimum sectional area, respectively. Both of them are obtained from measurements. The subscript “0” and “f” represent before and after the test.

Previous works [7–13] have calculated the stress triaxiality of round specimens with Bridgman’s formulation ((Eq 4)) which is a valid method to obtain the stress triaxiality over the minimum cross section accurately. Actually, the stress triaxiality of the specimen is not a constant value in the plastic strain process [18, 19]. The stress triaxiality changes with the geometry evolution of a specimen. What’s more, the stress triaxiality distributes not uniformly across the minimum sectional area. Thus, stress triaxiality is a complicated function of equivalent strain and space distribution. In the fracture model parameter fitting process, the computing method for determining the values of specimen stress triaxiality *η* corresponding to the fracture strain plays a decisive role to the fitting result. However, Bridgman’s formulation is not suitable for flat specimens either. For these reasons, a different technique is required to obtain a reasonable value of stress triaxiality. Bao and Wierzbicki [19] used a new concept of average stress triaxiality *η*_*av*_. The average stress triaxiality is interpreted by the integration of the stress triaxiality with respect to the equivalent plastic strain as shown in (Eq 17).

$$\eta_{av}=\frac{1}{\varepsilon_{f}}\int_{0}^{\varepsilon_{f}}\eta \;d\varepsilon_{p,eq}=\frac{1}{\varepsilon_{f}}\sum_{t=0}^{t=t_{f}}\eta_{t}{\left(\Delta \varepsilon_{p,eq}\right)}_{t}$$ (17)

where *t*_*f*_ is the time to fracture; *ε*_*p*,*eq*_ is the equivalent plastic strain. The average stress triaxiality is the summation of stress triaxiality multiplied by the incremental equivalent plastic strain at *t*_*f*_.

In this work, a method combining experimental data and numerical simulations was applied to obtain the stress triaxiality history along the entire experiment. The procedure is as follows:

Step 1: Compute numerical simulations of the tested specimens with a suitable non-linear finite element code. Confirm that the engineering stress-strain curves obtained from FE simulation and tests are coincident to each other. It should be noted that a fracture criterion is not used.

Step 2: Identify the engineering failure strain by comparing numerical and experimental curves. Find the time step of specimen fracture.

Step 3: Obtain stress triaxiality and equivalent plastic strain histories in the most critical elements, which may be defined as those for which the values of stress triaxiality and equivalent plastic strain are most unfavorable. One should note that there is an assumption for the original J-C calibration [17], stress triaxiality and equivalent plastic strain distribute homogeneously across the minimum cross sectional area.

Step 4: Obtain the equivalent plastic strain at fracture for the most critical elements.

Step 5: Once the history of stress triaxiality will be obtained, the initial stress triaxiality can be read. Calculate the average stress triaxiality with (Eq 17). Then, two sets of stress triaxiality, i.e. the initial and average, are obtained.

Step 6: Plot the stress triaxiality versus the equivalent plastic strain at fracture. Formulate the relation between the stress triaxiality and the equivalent plastic strain at fracture.

By using the above methodology, two sets of points in the stress triaxiality versus equivalent plastic strain space can be obtained. One of the sets is the lower fracture limit. And the other one is the proposed calibration point set. In fact, according to previous works [18–21], with the necking developing the stress triaxiality in the minimum section area is increasing. And high stress triaxiality contributes to early fracture. Therefore, the initial stress triaxiality is supposed to correspond to the lower fracture limit while the other set corresponds to the average stress triaxiality, which is closer to the actual situation. It should be emphasized that in the stress triaxiality versus plastic strain at fracture space, the initial stress triaxiality corresponds to the equivalent plastic strain at fracture calculated with (Eq 16) which is considered to be the average value of plastic strain at fracture along the minimum cross section. The reason of choosing the most unfavorable elements for obtaining the stress triaxiality and equivalent plastic strain histories, instead of the average stress triaxiality, is that fracture begins once some element has failed.

## Results

### Stress-strain curves at different strain rates

**Fig 6:**
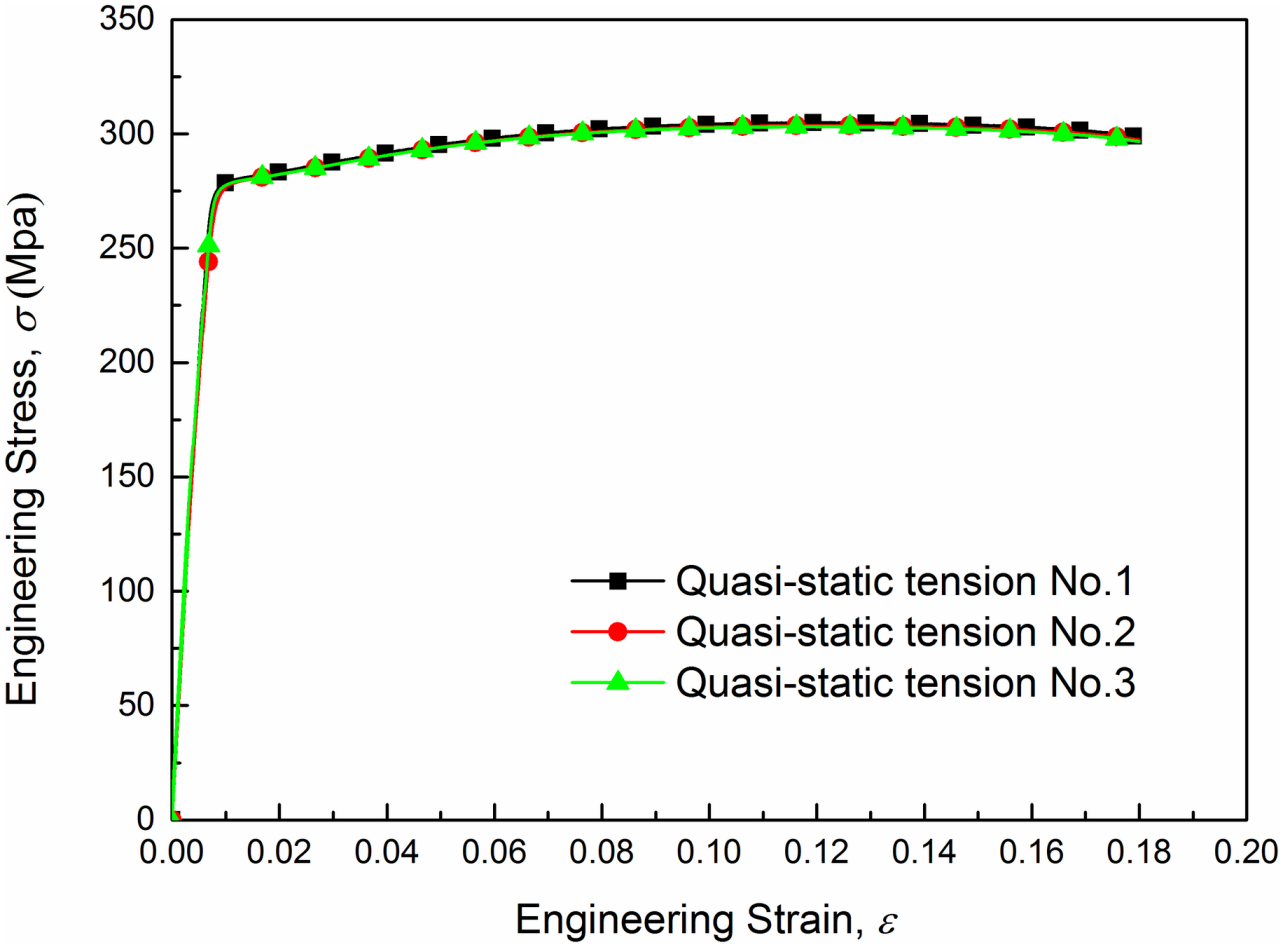
Engineering stress-strain curve under quasi-static tension.

The engineering stress-strain curves of AA6082-T6 under quasi tension are shown for 3 samples in Fig 6. In the beginning of tensile loading, the material shows linear elastic behavior within the elastic regime. When the stress reaches the yield limit (about 270 MPa) which means that the material comes into the plastic stage the variation of the stress tends to be much gentler till the stress meets ultimate strength. After yielding, further loading makes the material enter the plastic phase. A strain hardening effect can be seen in each case.

**Fig 7:**
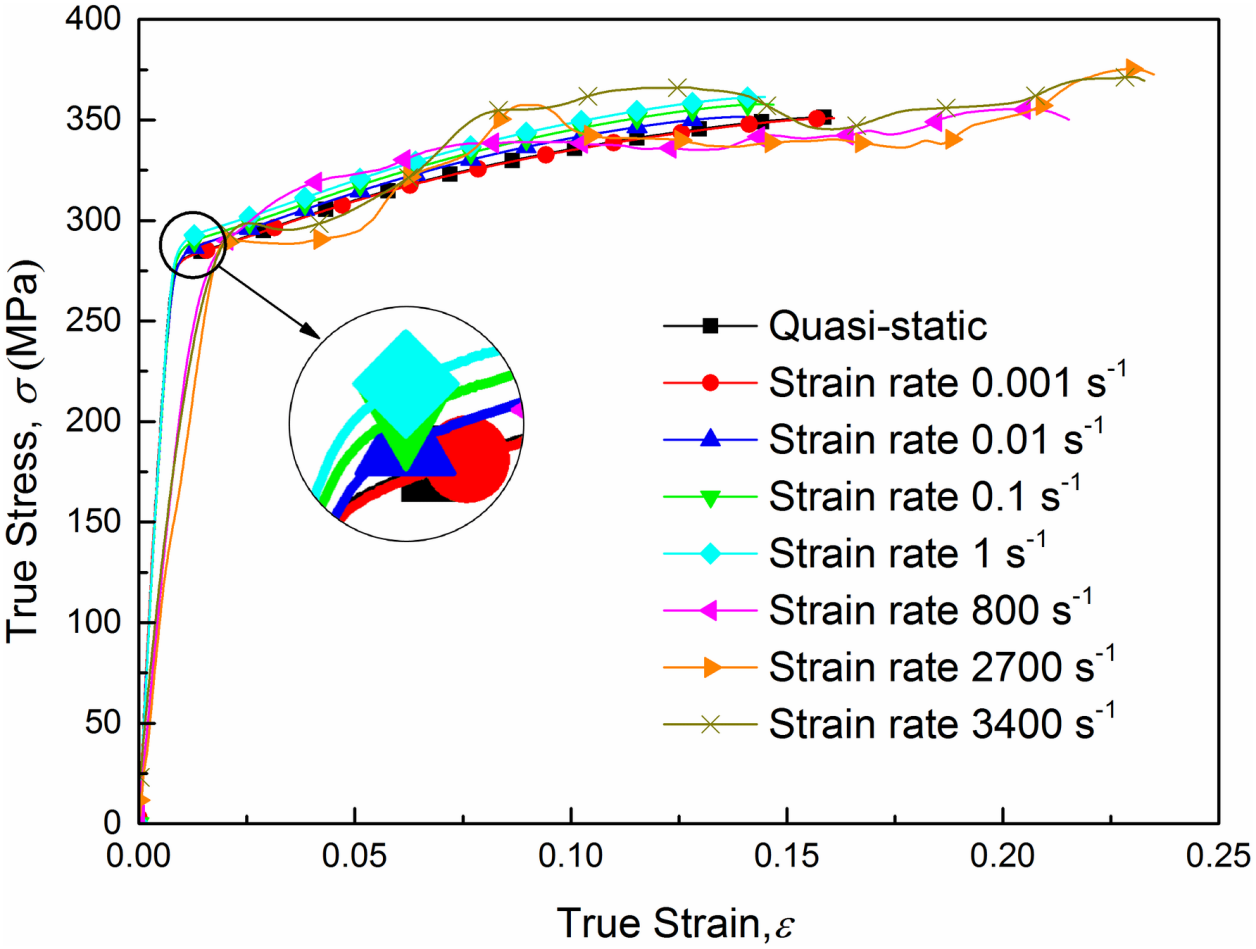
True stress-strain curves for quasi-static and dynamic tension tests at strain rates from 800s^-1^ to 3400s^-1^.

**Fig 8:**
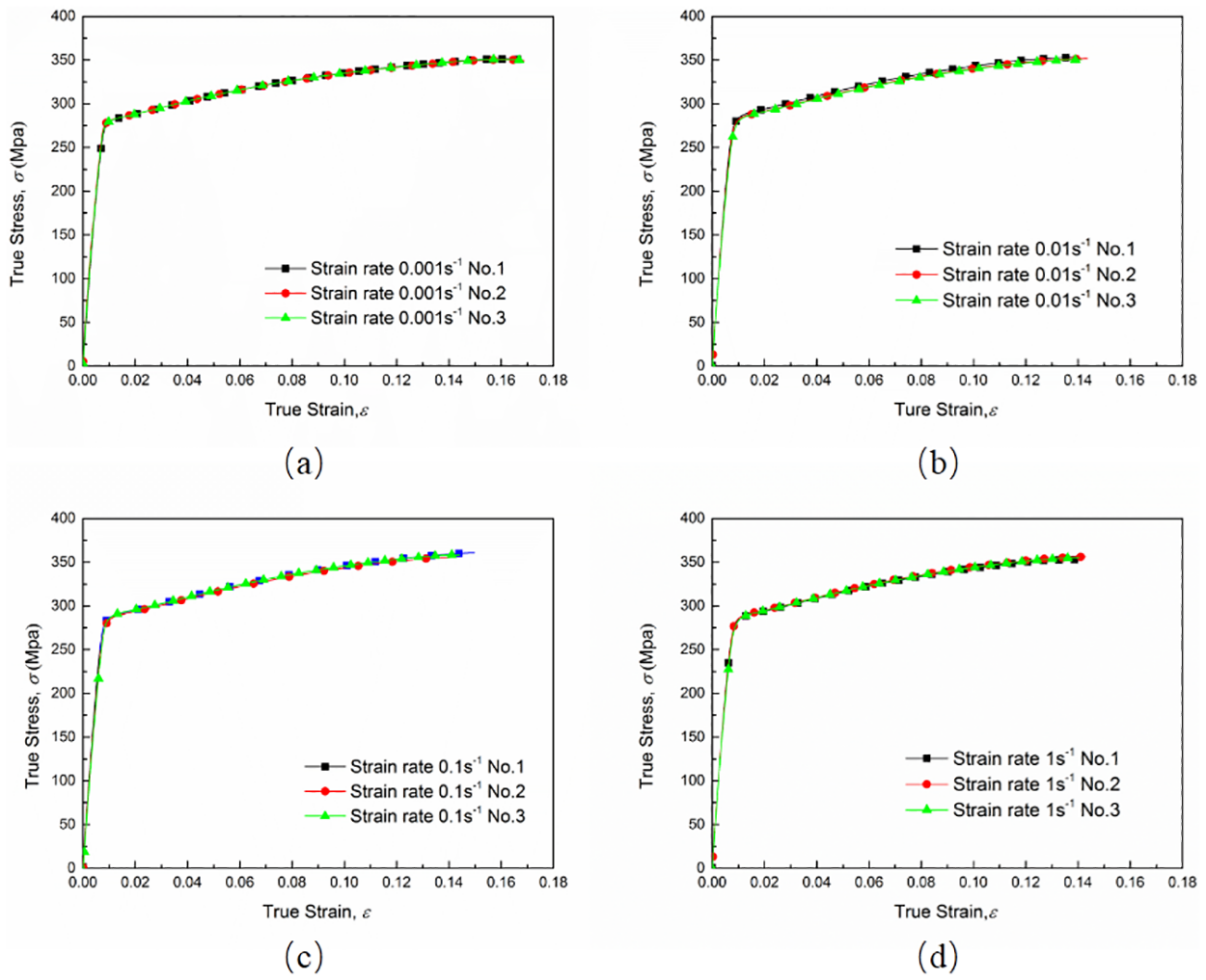
True stress-strain curves at strain rate (a) 0.001 s^-1^ (b) 0.01 s^-1^ (c) 0.1 s^-1^ and (d) 1 s^-1^.

Fig 7 shows true stress-strain curves of AA6082-T6 alloy at different strain rates under tension loading. The true stress and true strain are calculated using (Eq 2), which is valid up to necking. Curves in Fig 7 show that the ultimate strength appears in the final stage of deformation, which means that (Eq 2) is valid up to necking. The limit stresses are presented in Table 2. From a general view, no significant differences on the values of yield stresses and ultimate stresses can be observed between quasi-static tension tests and dynamic tests for this material during the plastic strain hardening phase. However, positive effects of strain rate on the yield stress can be clearly observed within the strain rate range from 0.001 s^-1^ to 1 s^-1^. Experimental data for AA6082-T6 indicated that an increase in strain rate from 0.001 s^-1^ to 1 s^-1^ gives about a 2.5% increase in yield stress, while the ultimate strength rises from 351.51 MPa to 370.99 MPa with an increase of 5.5%. And it is worth noting that curves for quasi-static tension and strain rate 0.001 s^-1^ are overlapped by each other completely which means that the strain rate sensitivity is approximately zero in this range. True stress-strain curves from strain rate 0.001 s^-1^ to 1 s^-1^ are plotted in Fig 8. As we can see, experimental data of three repeated tests for each strain rate agrees very well, which excludes the effect of experimental spread. Thus, a conclusion can be drawn that AA6082-T6 exhibits a kind of relatively weak strain rate sensitivity within strain rate range from 0.001 s^-1^ to 1 s^-1^ while for high strain rate (above 800 s^-1^) strain rate have little influence on the material’s plastic mechanical response. Based on the above point of view a strain rate related Johnson-Cook constitutive model is proposed in the sections below.

**Table 2:** Summary of mechanical properties under different strain rate tests.

Strain rate $\dot{\varepsilon }$ (s^-1^)	0.0001	0.001	0.01	0.1	1	800	2700	3400
Yield stress *σ*_*y*_ (MPa)	277.33	277.83	281.23	282.51	284.90	284.71	286.11	288.83
Ultimate stress *σ*_*u*_ (MPa)	351.51	350.83	351.58	357.79	361.37	355.44	375.49	370.99

### J-C constitutive model of AA6082-T6 alloy

**Fig 9:**
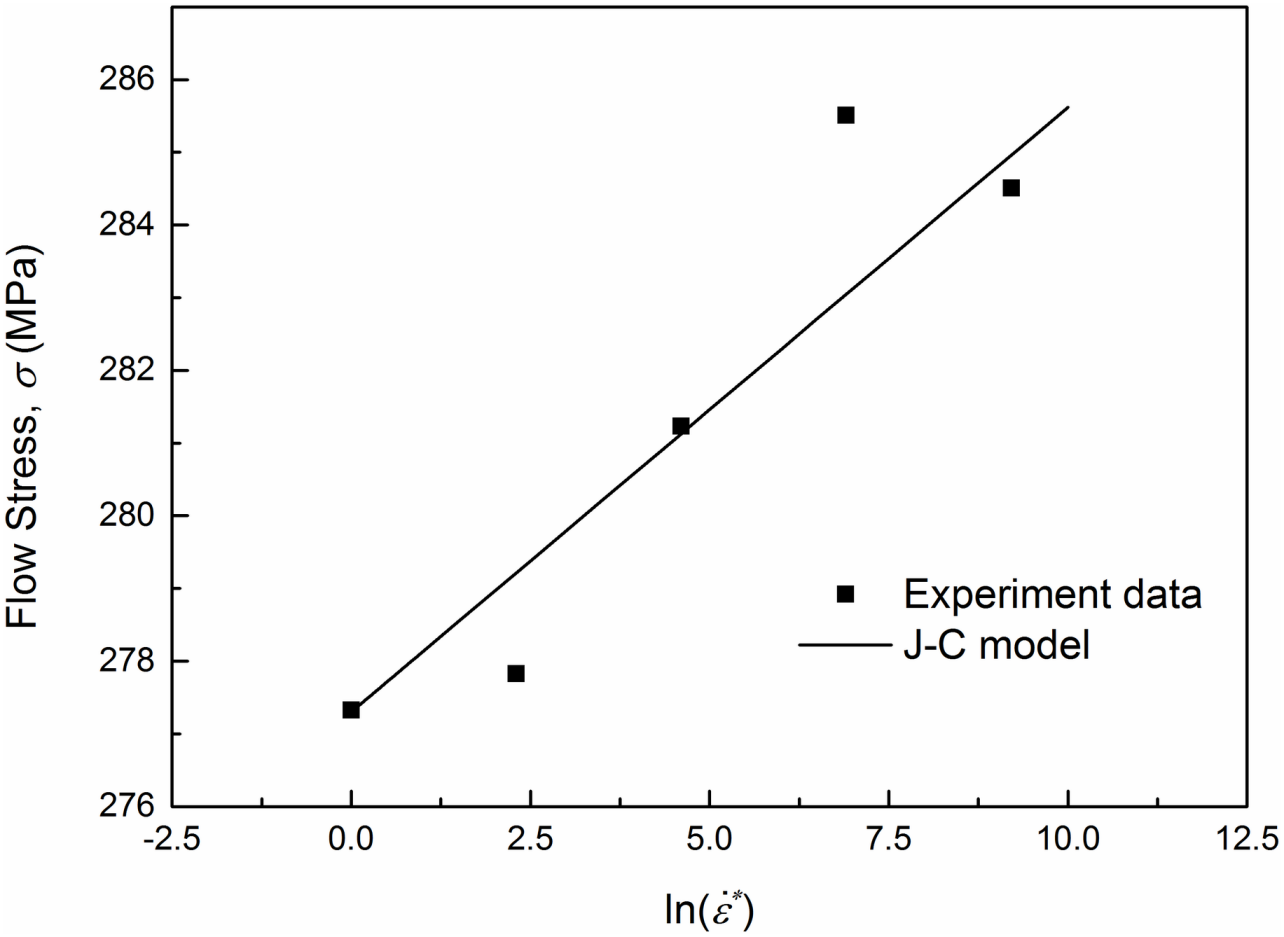
Flow stress comparison between the Johnson-Cook model and experimental results at low strain rates (0.0001~1 s^-1^).

**Fig 10:**
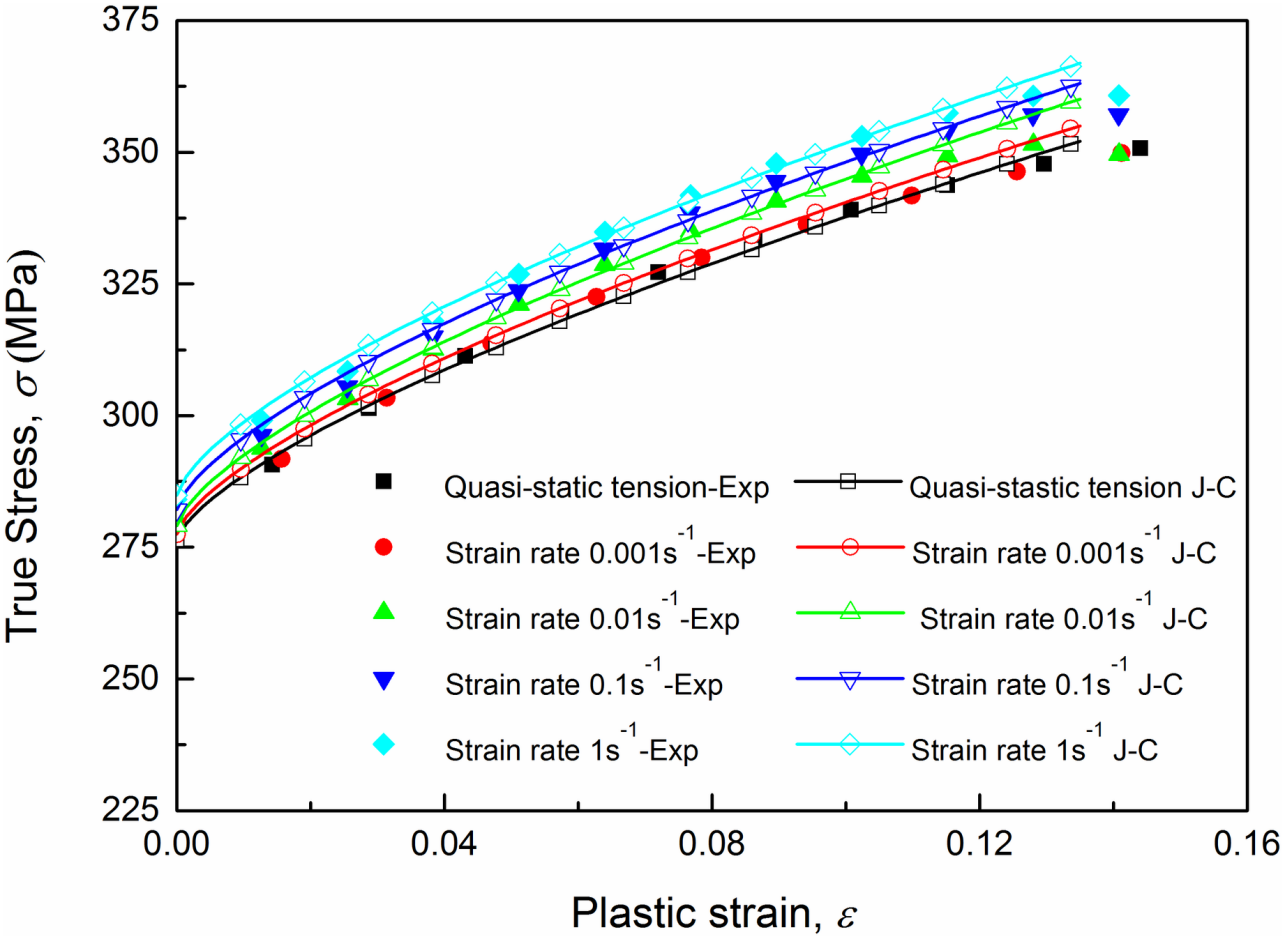
True stress-plastic strain comparison between experimental data and J-C model results at strain rates from 0.0001 s^-1^ to 1 s^-1^.

**Fig 11:**
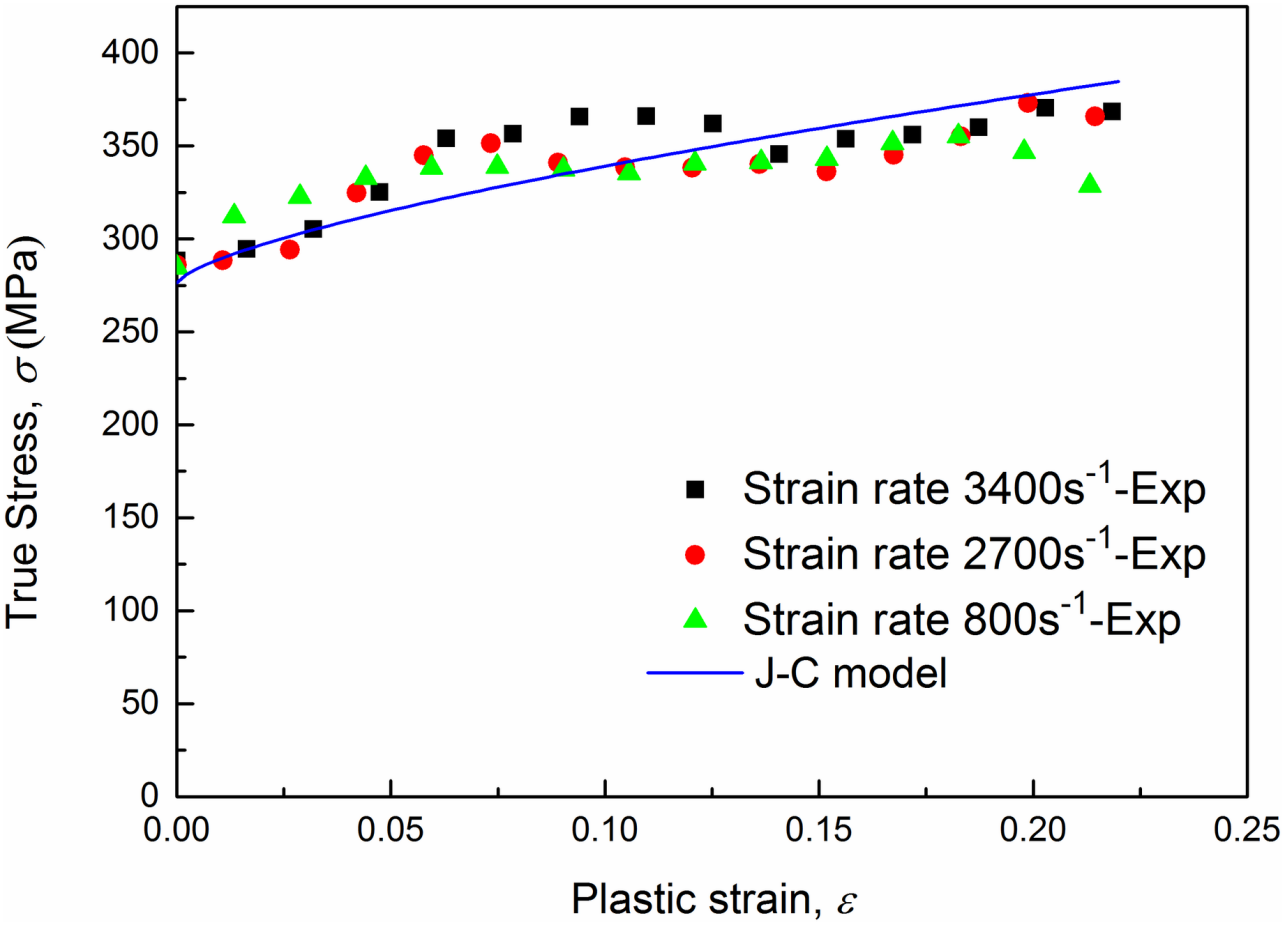
Comparison between stress-strain curves from experiments and the J-C model at strain rates 800~3400 s^-1^.

The constants of the Johnson-Cook model for the investigated material are listed in Table 3. In (Eq 9), let the strain *ε* equal zero then the flow stresses were obtained at low strain rates and shown in Fig 9. The results calculated by the J-C model are in good agreement with the experimental values, and verify its effectiveness. In Fig 9, the stress vs. strain rate experimental data point at strain rate 0.001 s^-1^ is compared with the value of the J-C model as well, and the difference in stress is less than 2 MPa and small enough to neglect. Thus, for convenience the strain rate sensitivity range of the investigated material can be expanded to the range from 0.0001 s^-1^ to 1 s^-1^. The J-C model is used to predict the yield stresses under both low and high strain rates. The predicted results are listed in Table 4 and compared with the experimental results ($\text{Relative error }=\;\left|\frac{\sigma_{y}^{J-C}-\sigma_{y}^{exp}}{\sigma_{y}^{exp}}\right|\times 100\%$). It can be found that the yield stresses given by the J-C model at high strain rates are almost the same due to the small value of the rate sensitivity coefficient. And the three predicted values are all greater than the experimental results. This is because the loading process at a high strain rate increases the temperature and softens the specimens. The temperature increase can be computed with (Eq 6). The coefficient *m* can be determined with the least squares method. The parameters used in (Eq 6) and the value of *m* are listed in Table 5. The stress-strain curves at low and high strain rates obtained from the Johnson-Cook model are compared with those of experiments in Figs 10 and 11 respectively. The fitting deviation coefficient *R*^2^ is 0.9998 in the linear fitting process of Fig 10. Comparison in Fig 10 shows excellent agreement between the experimental and predicted data by the proposed J-C model at low strain rates. For high strain rate tension, the three predicted curves are almost completely overlapped due to the small value of rate sensitivity coefficient as well. In general, the predicted curves can represent the actual plastic stress. In conclusion, the J-C model is able to describe the flow behavior of AA6082-T6.

**Table 3:** The Johnson-Cook model parameters for the investigated material.

Parameters	*A* (MPa)	*B* (MPa)	*n*	*C*	${\dot{\varepsilon }}_{0}$(s^-1^)
Value	277.33	307.93	0.69	0.0032	0.0001

**Table 4:** Comparison between yield stresses from experiments and the J-C model.

Strain rate $\dot{\varepsilon }$ (s^-1^)	0.0001	0.001	0.01	0.1	1	800	2700	3400
Experimental yield stress $\sigma_{y}^{exp}$ (MPa) J-C model yield stress $\sigma_{y}^{J-C}$ (MPa)	277.33	277.83	281.23	282.51	284.90	284.71	286.11	288.83
	277.33	279.25	281.16	283.08	284.99	290.55	291.57	291.76
Relative error (%)	0	0.511	0.025	0.205	0.032	2.050	1.908	1.010

**Table 5:** Values of material parameters and temperature coefficient.

Parameters	*ρ* (g/cm^3^)	*C*_*p*_ (J·(K·g)^-1^)	*χ*	*m*	E (MPa)	*μ*
Value	2.71	0.96	0.9	1.28	71000	0.3

### Numerical simulations of smooth and notched specimens

In this paper, a non-linear finite element code, ABAQUS/Standard and ABAQUS/Explicit were both used for the numerical simulations under quasi-static and low strain rates respectively. Four smooth and notched specimen models were simulated. The J-C constitutive relation built above was used to define isotropic elastic-plastic material property. Other parameters of material property are referred to Table 5. It is notable that no failure criterion is introduced due to the calibration aim of simulation instead of validation.

**Fig 12:**
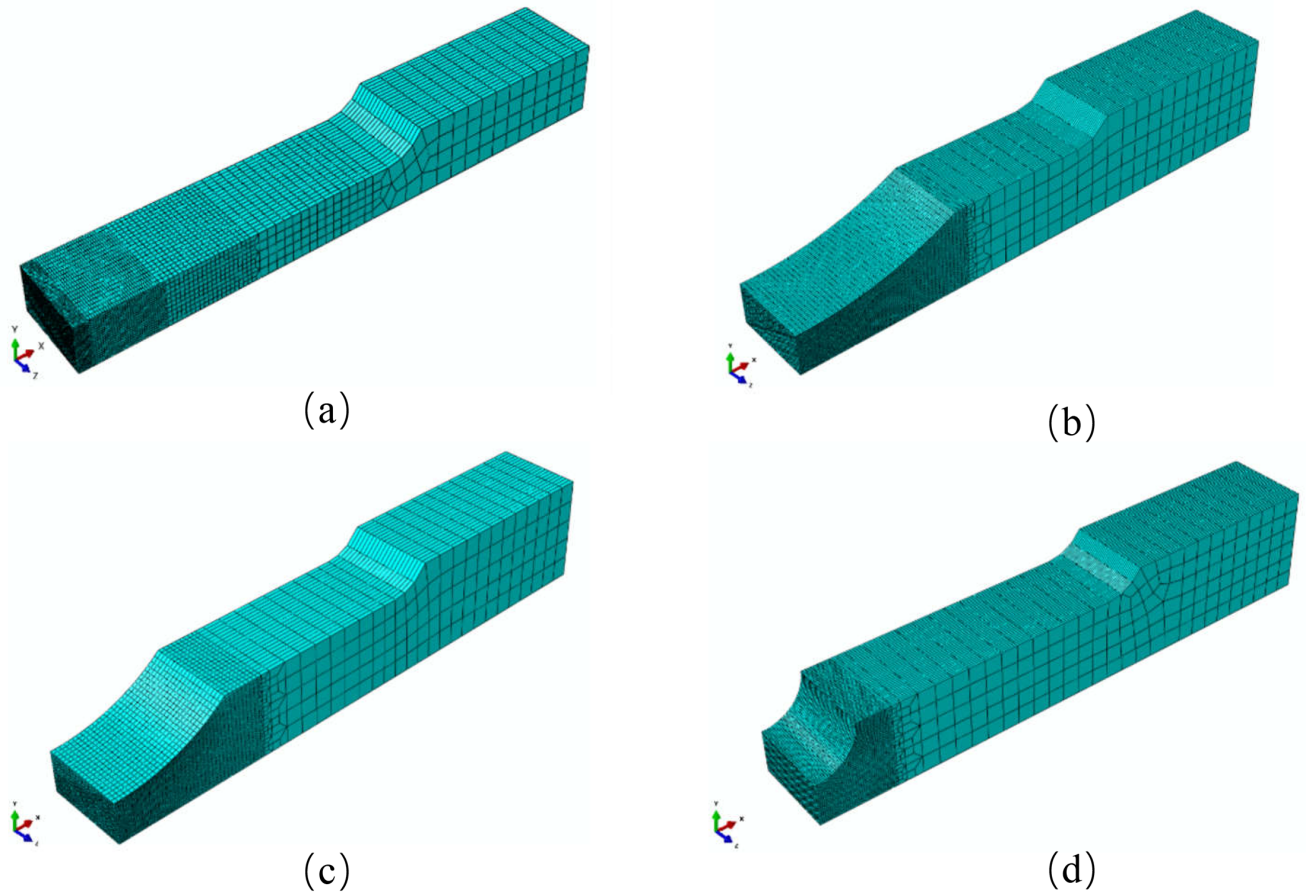
Finite element model meshes of (a) smooth and notched specimens with notch radius of (b) 90 mm, (c) 40 mm and (d) 10 mm for ABAQUS/Standard.

**Fig 13:**
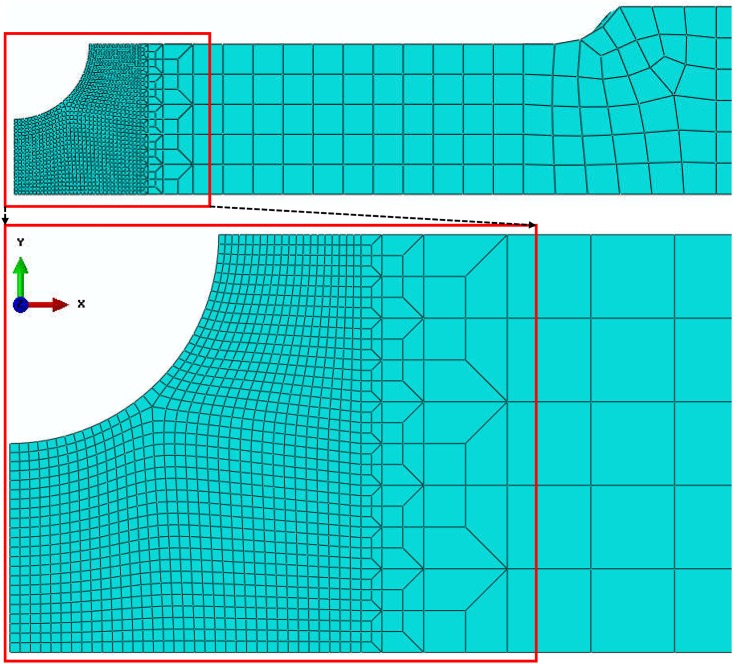
Typical finite element mesh notched specimen model with a notch radius of 10 mm.

Only the 1/4 of each of the specimens is modeled to reduce the computing time. The element type is the three dimensional hexahedral element with reduced integration (C3D8R). As with the experiments, axial velocity loads are applied at the end of the finite element models. The meshes of the finite element models are shown in Fig 12. Mesh refinement technique is adopted near the fracture zone. Fig 13 shows a typical finite element mesh of a notched specimen model with the notch radius of 10 mm. The mesh of the smooth specimen has 119120 elements while the specimens with notch radius *R* = 90 mm, *R* = 40 mm and *R* = 10 mm have 75400, 34000 and 54440 elements, respectively.

**Fig 14:**
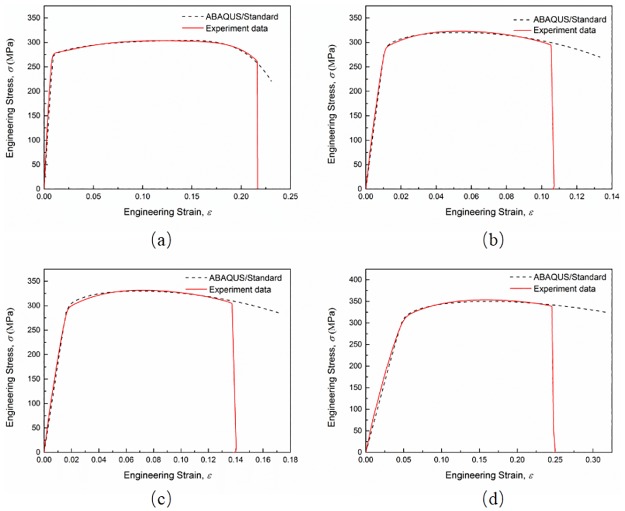
Engineering stress-strain curves from experiments and ABAQUS/Standard numerical simulations for (a) smooth specimen, (b) 90 mm, (c) 40 mm and (d) 10 mm notch radius specimens.

**Fig 15:**
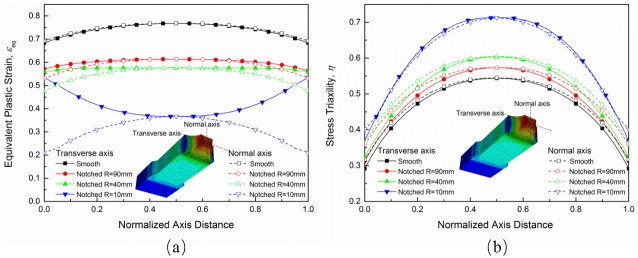
(a) Stress triaxialities and (b) equivalent plastic strain profiles of smooth and notched specimens at the minimum cross section at the time of fracture.

The engineering stress-strain curves from numerical simulations agree well with those from experiments as shown in Fig 14. By simulating the processes of tensile tests, the spatial distribution of the stress triaxiality and the equivalent plastic strain from the beginning to the fracture can be obtained. Fig 15 shows stress triaxiality and equivalent plastic strain profiles of smooth and notched specimens along two perpendicular axes at the minimum cross section. Each of the profile is obtained from ABAQUS/Standard numerical simulations at the step time of fracture. The stress triaxiality values at the minimum cross section decrease from the center of the rectangular cross section to the edge. However, the stress triaxialities of the edges for different specimens are in a rough range from 0.33 to 0.4 where the difference is not as marked as the difference at central area. In the view of the space, the plastic deformation concentrates nearby the minimum section area, and the maximum value of the stress triaxiality is located in the center of the minimum section area almost throughout the whole plastic strain stage, as expected. And the stress triaxiality values along two perpendicular axis show a small difference. The equivalent plastic strain shows the same tendency as the stress triaxiality. Nonetheless, as shown in Fig 15(b), the distribution of equivalent plastic strain along the transverse axis of 10 mm notch radius specimen exhibits an opposite behavior, which is opposite with the variation along the transverse axis too and may lead to an advanced failure. After all, due to the marked difference of the stress triaxiality level between the cross section center and the notch surface along the transverse axis for the 10 mm notch radius specimen, failure is supposed to begin at the center of the minimum cross section. In conclusion, the elements that failure begin with located in the center of the minimum cross sections of the specimens.

**Fig 16:**
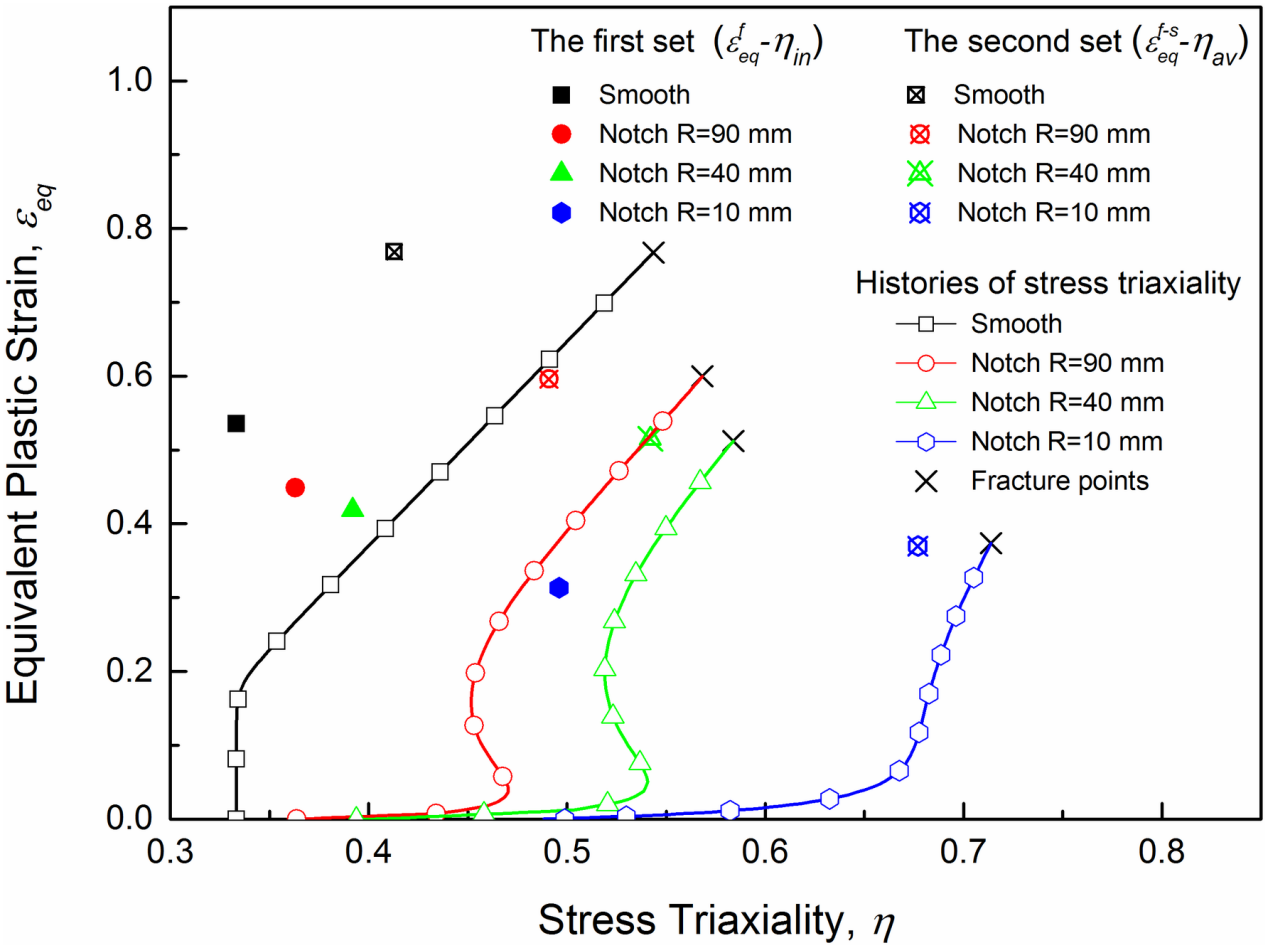
The two sets of points and histories of the stress triaxiality in quasi-static tensile tests.

The two sets of points in the equivalent plastic strain verse the stress triaxiality space are plotted in Fig 16. The first set of points given by a common method, in which the initial stress triaxiality (denoted as *η*_*in*_) and the fracture strain calculated with (Eq 16) were adopted. All the fracture area ratios (*A*_0_/*A*_*f*_) of minimum sectional area and the calculated fracture strain $\varepsilon_{eq}^{f}$ are listed in Table 6 as well as the fracture strains from numerical simulations $\varepsilon_{eq}^{f-s}$, and the data are all averaged results of repeated tests. With the same height and width at the minimum cross section, *a*_0_ and *b*_0_ are both 20 mm for each specimen. And the second set is the averaged stress triaxiality versus fracture strain obtained from ABAQUS/Standard numerical simulation. Compared with the latter set points the points obtained by the common way incline to locate below, which implies a more conservative fracture criterion. Stress triaxiality histories of the critical elements are plotted as well. Curves in Fig 16 exhibit a generally increasing tendency with the increase of plastic strain for each type of the specimens. This tendency is more conspicuous for specimens with larger initial stress triaxialities.

**Table 6:** Fracture reduction ratio (*A*_0_/*A*_*f*_) and comparison of equivalent plastic strains to fracture between calculated results from (Eq 1^6^) and numerical simulations.

Specimen types	Test strain rate (s^-1^)	*A*_*f*_ (mm^2^)	*A*_0_/*A*_*f*_	$\varepsilon_{eq}^{f}$	$\varepsilon_{eq}^{f-s}$
Smooth	Quasi-static	217.984	1.835	0.607	0.768
	0.001	234.055	1.709	0.536	0.612
	0.01	232.558	1.702	0.532	0.585
	0.1	243.457	1.643	0.499	0.553
	1	244.051	1.639	0.494	0.545
Notched R = 90 mm	Quasi-static	255.265	1.567	0.449	0.59977
Notched R = 40 mm	Quasi-static	263.158	1.520	0.419	0.51229
Notched R = 10 mm	Quasi-static	292.398	1.368	0.313	0.37395

$\varepsilon_{eq}^{f-s}$: The equivalent plastic strain at fracture obtained from numerical simulations.

### Johnson-Cook fracture model constants identification

The construction of the J-C fracture model implies that five constants must be identified (see (Eq 10)). In present work, the effect of temperature is not taken into consideration. Thus, the constant in the temperature-dependent factor is neglected, then the formula, with four constants left to identify, is simplified as (Eq 9). The calibration of the J-C fracture model is performed using the data set of the average stress triaxiality versus equivalent plastic strain at fracture while the other J-C fracture model (the lower limit) is built using the data set of the initial average stress triaxiality versus equivalent plastic strain at fracture.

Similar parameter fitting processes have been done by minimizing the residuals with success [5–7]. In the present work, the same method is adopted. The quasi-static tensile test data of smooth and notched specimens is used to fit the first three constants, *D*_1_, *D*_2_ and *D*_3_. (Eq 9) can be rewritten in such case just with the first factor:

$$\varepsilon_{f}=D_{1}+D_{2}\text{exp}\left(D_{3}\eta \right)$$ (18)

J-C fracture model parameters for the common method and calibration are listed in Table 7.

**Table 7:** J-C fracture model parameters for the common method and calibration.

Parameter	*D*_1_	*D*_2_	*D*_3_
Common method	0.26862	9.20158	-10.6552
Calibration	0.21125	3.91116	-4.72526

## Discussion

### Dynamic mechanical response

Tests on aluminum alloy strain-rate sensitivity are reported by a large number of researchers. Many studies [3, 22, 23] have investigated the stress-strain behavior of AA6xxx and other aluminum alloys at a wide strain rate range. Compared with others AA6xxx aluminum alloys exhibit a rather low rate sensitivity, while AA7xxx alloys show moderate rate sensitivity. However, AA6005-T6 shows a rather strong rate sensitivity according to [24]. Actually, tests on AA6082-T6 aluminum alloy strain-rate sensitivity have been studied by Chen et al. [3]. In this paper, a series of tensile tests on AA6082-T6 smooth specimens in the strain rate range from quasi-static to 3400s^-1^ were conducted, and the stress-strain curves are plotted in Fig 7. A similar result is obtained, that is, little rate sensitivity is observed on the mechanical response of AA6082. However, an additional phenomenon is observed that a slight but clear rate sensitivity exists in the strain rate range from quasi-static to 1s^-1^, while the rate sensitivity is not found in dynamic tensile tests. The phenomenon is considered to be caused by the adiabatic heating of specimens at high strain rates. Such rate insensitivity or even negative sensitivity feature is an unfavorable property because it contributes to a faster fracture after onset of plastic deformation localization. Actually, plastic deformation is a process that concentrates in the localization zone which is a relatively small region. This concentration may increase the strain rate locally. In general, increased strain rate implies high flow stress, stresses can thus redistribute in the necking zone. However, for a material featuring negative rate sensitivity, fracture may take place prematurely because the softening effect prevents the redistribution.

### Validation of the J-C fracture model

**Fig 17:**
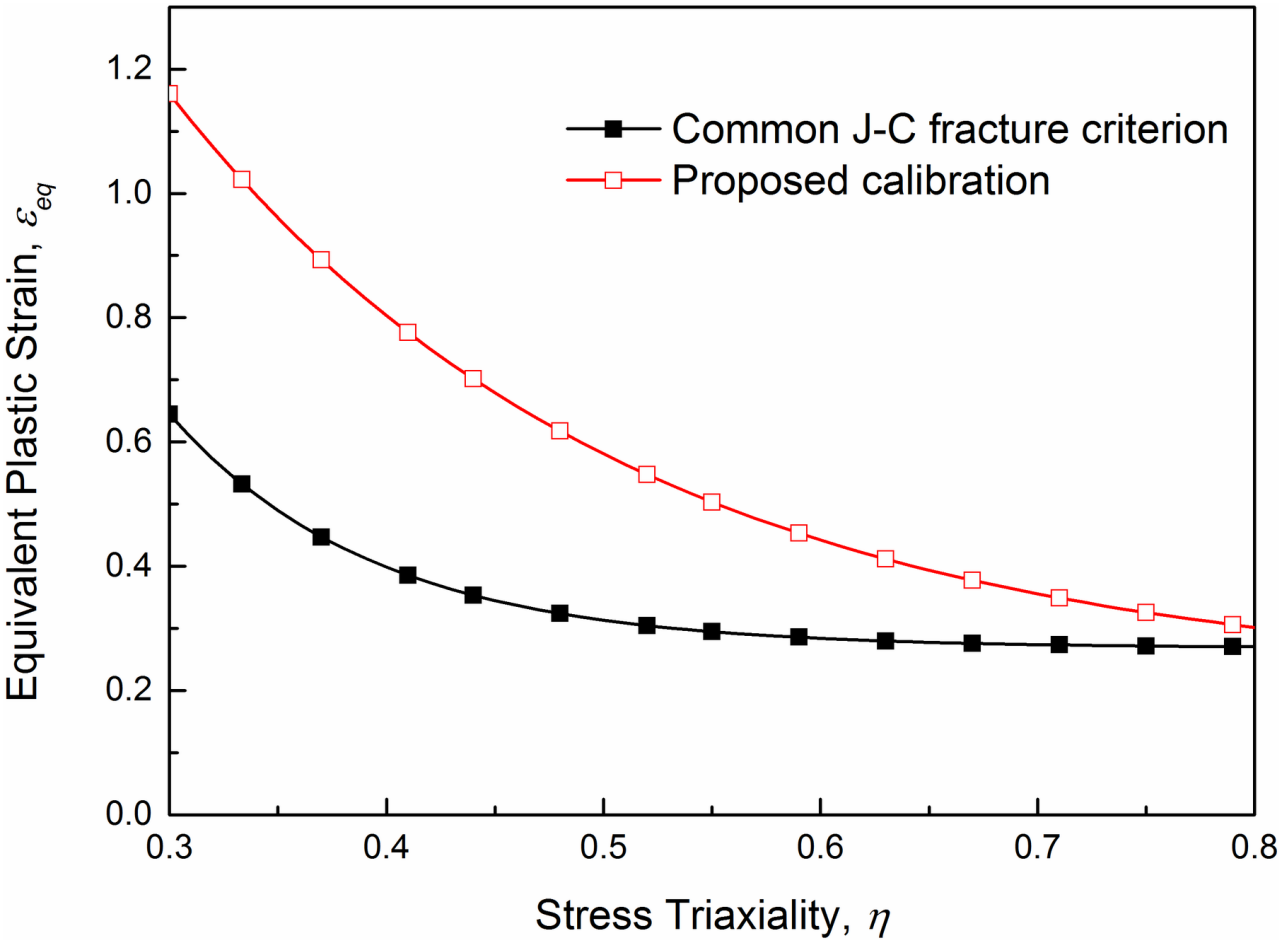
Comparison between the common J-C fracture model and the proposed calibration.

In this paper, a calibration methodology is proposed to obtain a simplified J-C fracture model to describe the ductile fracture more accurately. Previous works have built J-C fracture models for various materials with success. A common method is used to obtain the data set of stress triaxialities and equivalent plastic strain at fracture that take into account the initial stress triaxiality (given by Bridgman’s analysis (Eq 4)) and the equivalent plastic strain at fracture calculated with (Eq 14). However, this method may lead to a relatively conservative result due to the underestimate of global stress triaxialities. For this reason, some calibrations for the J-C fracture model were proposed. Bao et al. [20] proposed to integrate the stress triaxiality of the entire process with respect to the equivalent plastic strain as shown in (Eq 15). Such a method is used by Choung et al. [25, 26] to estimate the failure strain of EH36, and a good prediction was provided. Erice et al. [19] suggested an approach by averaging two data sets, the “lower” and the “upper” fracture limits. They took the data set from common method as the “lower” limit, and obtain the “upper” data set from numerical simulations where the stress triaxialities and the equivalent plastic fracture strains of the critical elements at the time step of fracture were used. Such calibration by merely averaging the two limits seems closer to reality than the common method, however, the accuracy may not be good enough. Here, the two methods were combined to calibrate the J-C fracture model. The average stress triaxiality is used and the equivalent plastic fracture strains are obtained from numerical simulations at the time step of fracture. Fig 17 shows the two models in the stress triaxiality versus equivalent plastic strain space. What is noteworthy is that the relation is given only in the region of high stress triaxialities due to the limitation of J-C fracture model [20].

**Fig 18:**
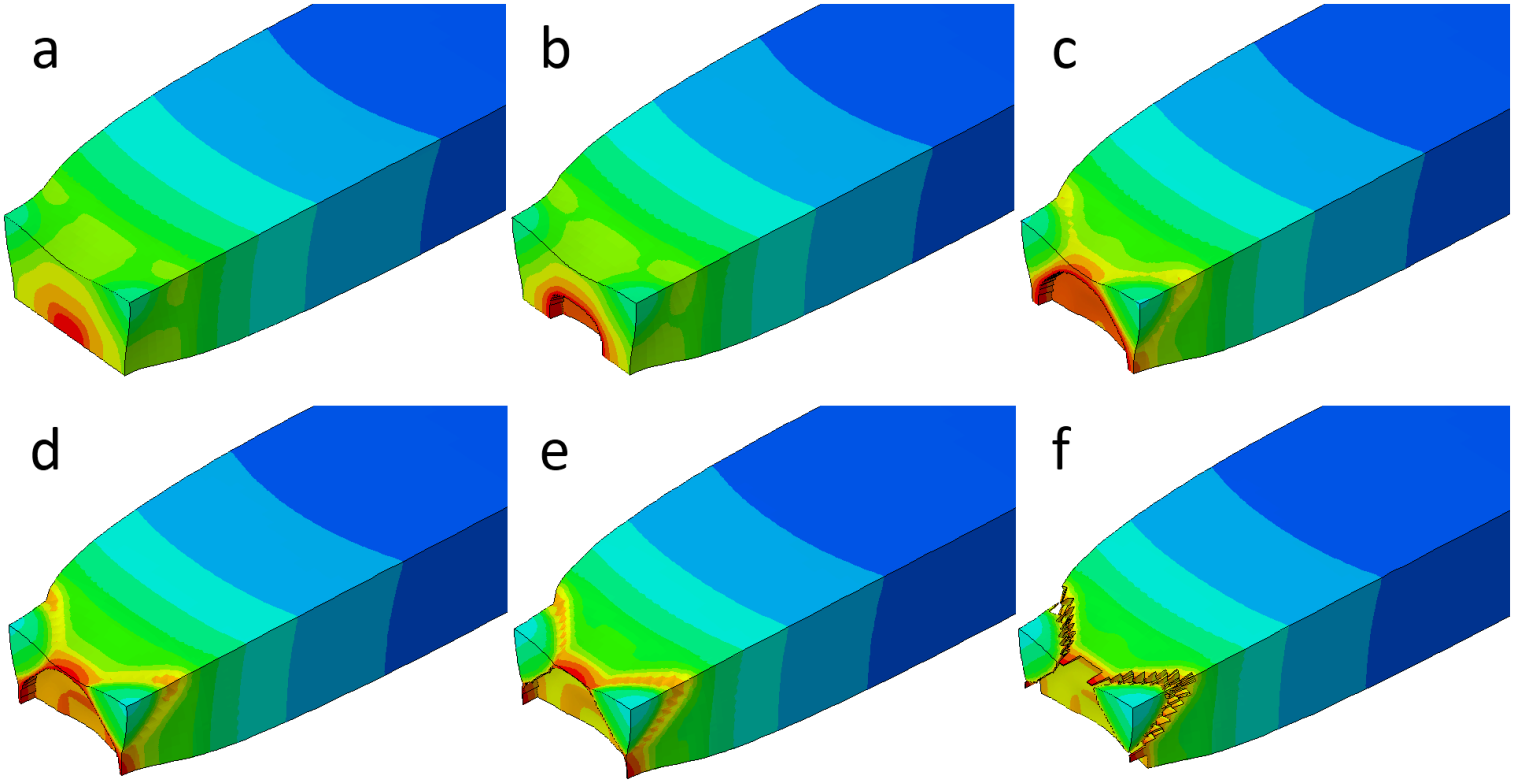
The simulated fracture process of a smooth specimen.

The calibrated J-C fracture model was used in the numerical simulations of the quasi-static tensile tests for smooth and notched specimens. Also, the J-C fracture model was used in ABAQUS/Explicit simulations for comparison. Fig 18 shows the fracture process of the smooth specimen in the numerical simulation. The fracture simulation gives a depiction of the stress triaxiality distribution and fracture process. When the equivalent plastic strain reaches the fracture strain elements are deleted automatically. It can be seen that failure started in the center area of the minimum cross section due to the high level of the stress triaxiality and proceeded towards the edge, then the corners were sheared to fracture at last.

**Fig 19:**
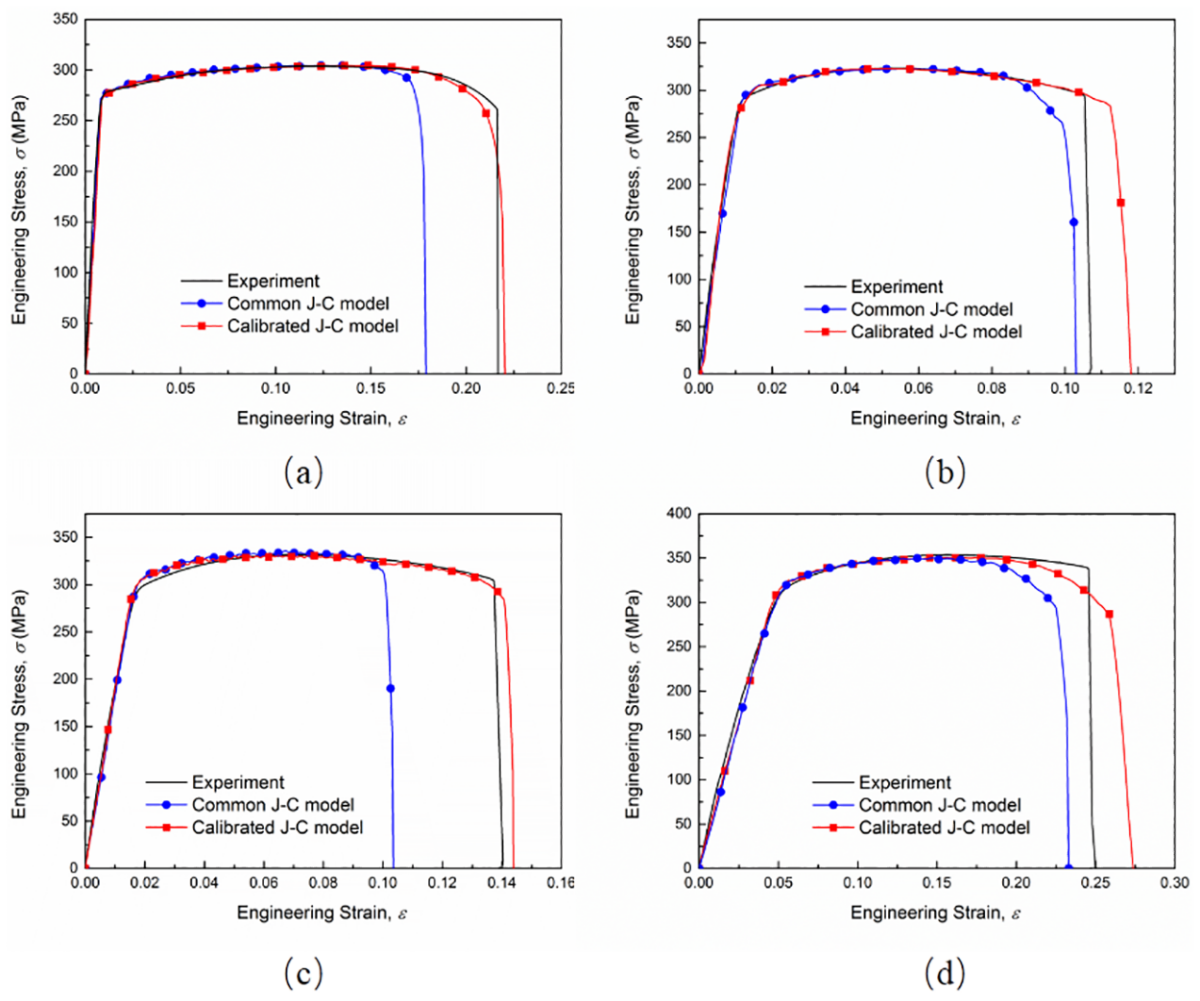
Engineering stress-strain curves from experiments and ABAQUS/Explicit numerical simulations for (a) smooth specimen, (b) 90 mm, (c) 40 mm and (d) 10 mm notch radius specimens.

The engineering stress-strain curves of smooth and notched specimens at quasi-static strain rate, obtained from experiments and numerical simulations respectively, are plotted in Fig 19 to validate the calibration of the J-C fracture model. The common J-C fracture model shows a relatively “conservative” estimating on the ductility of AA6082-T6 alloy as expected. The underestimation reaches to an averaging extent of 13.50%. And the average error of the engineering fracture strain after calibration is 6.05% which is a great improvement on fracture strain estimation. The results give an evidence that the calibration methodology can improve the accuracy of the J-C fracture model effectively.

### Charpy impact test

**Fig 20:**
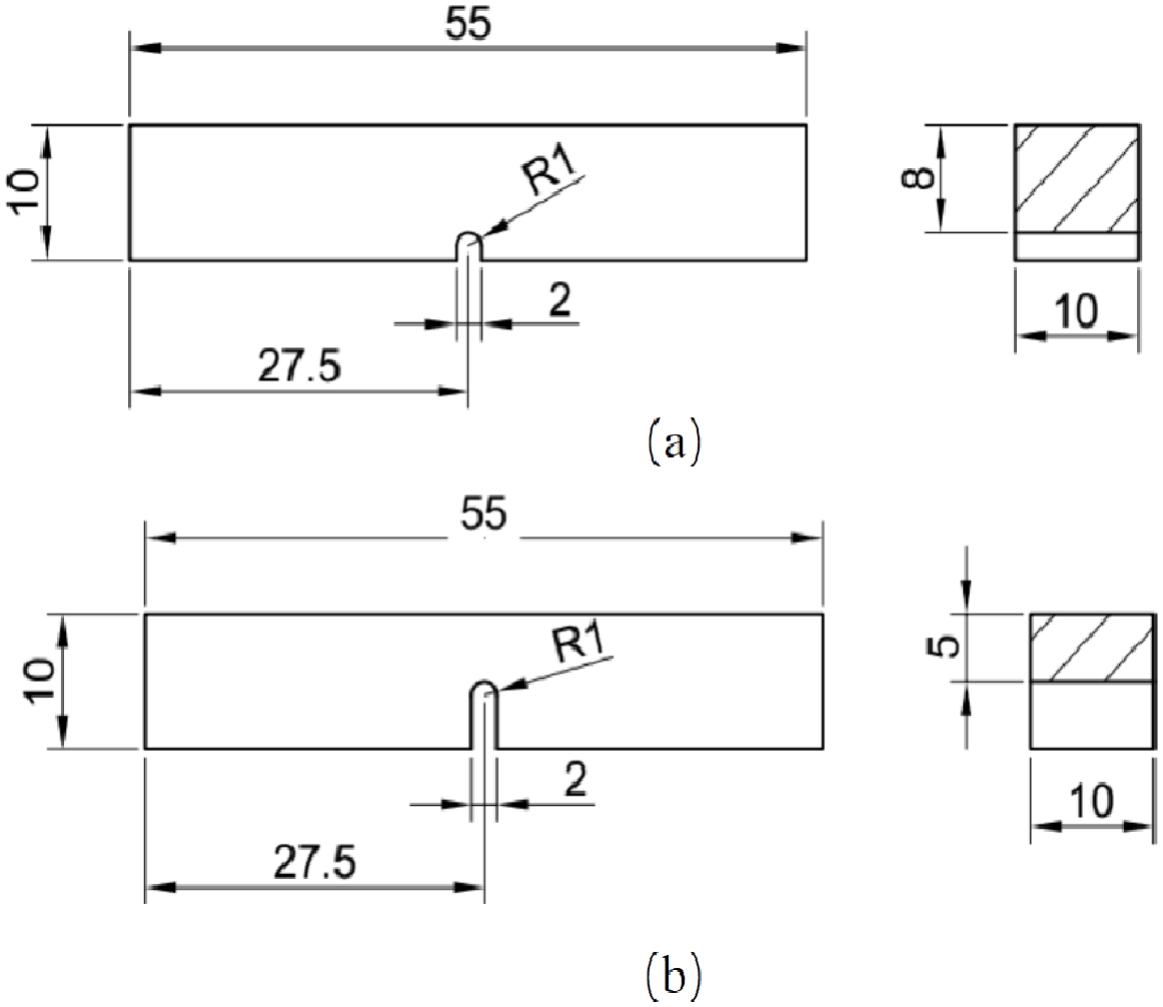
The dimensions of (a) 2 mm and (b) 5mm notch depth specimen in Charpy impact test.

Railway collision accidents are rare, but once it happened the consequences could be catastrophic. In recent years, researchers such as Peng et al. [27] have investigated the collision performance of train energy absorbing structures. AA6082-T6, as a train body manufacturing material is supposed to be of good impact resistance. To further investigate its dynamic impact fracture property, two groups of Charpy impact tests are carried out. Two groups of specimens with the same size but different notch depth are machined. The dimension is 55×10×10 mm as shown in Fig 20 (unit: mm). The pendulum impact testing machine provides 150 J impact energy by lifting the pendulum to a height, then released to impact the specimen placed horizontally bellow the pendulum. During the impact, part of the mechanical energy is dissipated by specimen deformation and fracture. The height of pendulum after impact is recorded, and the energy difference before and after the impact can thus be calculated.

**Fig 21:**
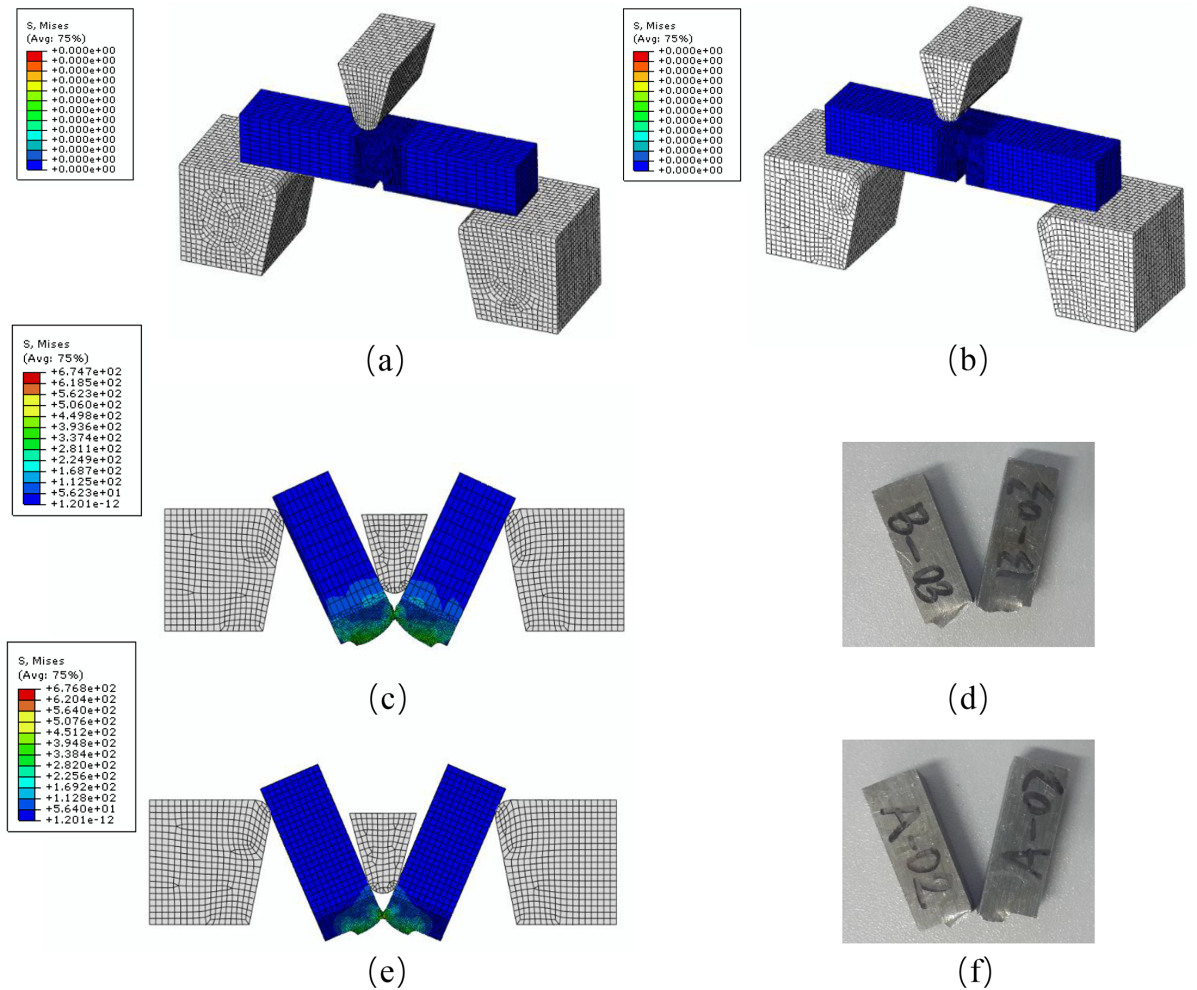
The FE models for (a) 2 mm and (b) 5mm notch depth specimen in Charpy impact test. The final deformation morphologies are compared with (c) simulation and (d) experiment results of 2 mm notch depth specimen as well as (e) simulation and (f) experiment results of 5 mm notch depth specimen.

Two finite element models corresponding to the two tests are established based on ABAQUS/Explicit to verify the effectiveness of the calibrated J-C fracture model. The material property is defined same as the models built above. Both of the common and calibrated J-C fracture model are used to simulate the tests. The models before and after the impact are shown in Fig 21. The deformation shape in simulation qualitatively agrees with the experiment results. On ther other hand, the Charpy impact energy obtained directly from experiment and that from the simulation are compared in Table 8. The energy absorption differences between simulation and experiment are 16.34% and 24.38% using the common J-C fracture model for specimens with the notch depth of 2 mm and 5 mm, respectively, which are much higher than them (4.91% and 11.14%) using the calibrated J-C fracture model. It indicates that fracture behavior of AA6082-T6 alloy can be more effectively described through the calibrated J-C failure model.

**Table 8:** Comparison of Charpy test results between experiment and simulation.

Notch depth	Experiment (J)	Simulation (J)	Difference (%)
2 mm	22.27	43.26 (common J-C)	16.34
		54.25 (calibrated J-C)	4.91
5 mm	51.71	16.84 (common J-C)	24.38
		19.79 (calibrated J-C)	11.14

### Fractography analysis

**Fig 22:**
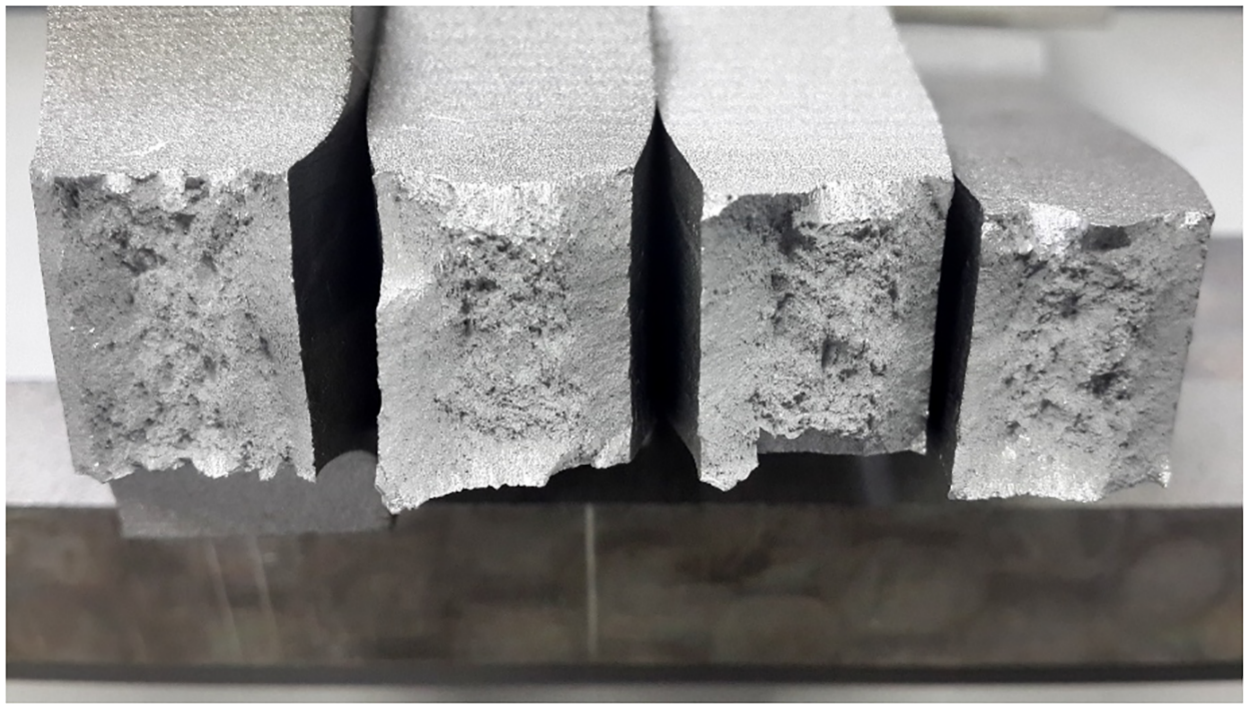
Notched specimens (notch radius 10 mm, 40 mm, and 90 mm from left to right) and smooth specimen after fracture tested at a quasi-static strain rate.

**Fig 23:**
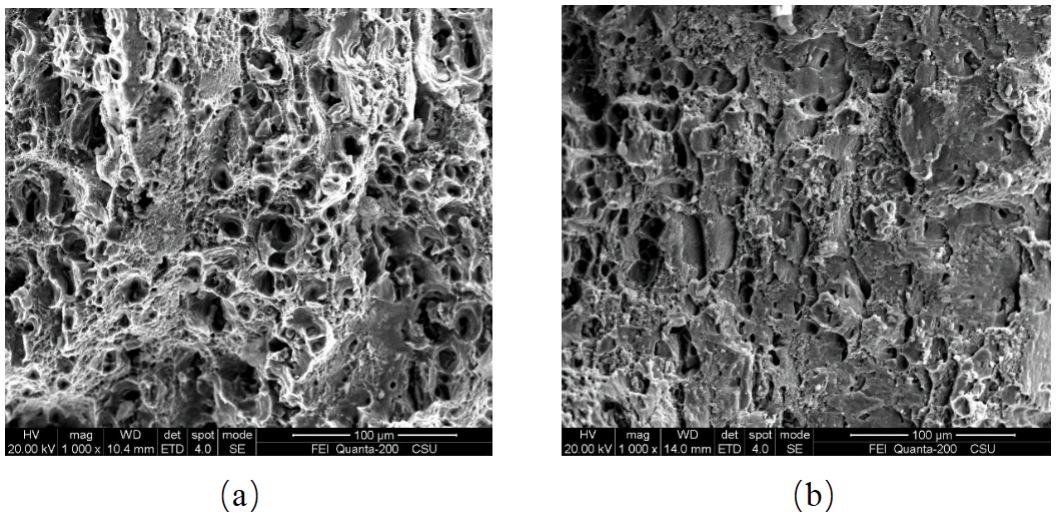
(a) Dimple fracture and (b) shear fracture on the fracture surface of the smooth specimen.

From the results of a series of tensile tests, it is found that AA6082-T6 exhibits an excellent ductility, with an elongation at fracture of 10% to 25%. A number of studies have been done on ductility damage and ductility fracture [28–31]. It is common view that the dimple fracture (experiencing the process of nucleation, growth, and coalescence of voids) and shear fracture (developed from shear band localization) are two primary fracture forms for ductile materials. Fig 22 shows the smooth and notched specimens after fracture at quasi-static strain rate. Typical cup-and-cone form fracture surfaces are shown. The central zone of the fracture surface shows ductile fracture full of bumps and hollows while the residuary area represents shear fracture with the surface along the maximum shear stress plane, at an angle of 45 degree to the tensile axis. SEM fractographs of the smooth specimen fracture surface are shown in Fig 23. Fig 23(a) shows a typical nucleation-growth-coalescence process of ductile fracture, where a lot of voids and dimples can be observed. In the central zone, the stress triaxiality is supposed to be higher than at the area close to the specimen surface. When a ductile material is subjected to hydro static stress, micro-voids nucleate in the central zone giving priority to impurities and second-phase particles, then grow bigger with the development of deformation, at last coalescence of grown void leads to many micro cracks. When the micro cracks link up and expand to the area close to the specimen surface shear fracture takes place. Fig 23(b) shows shear fracture mode.

**Fig 24:**
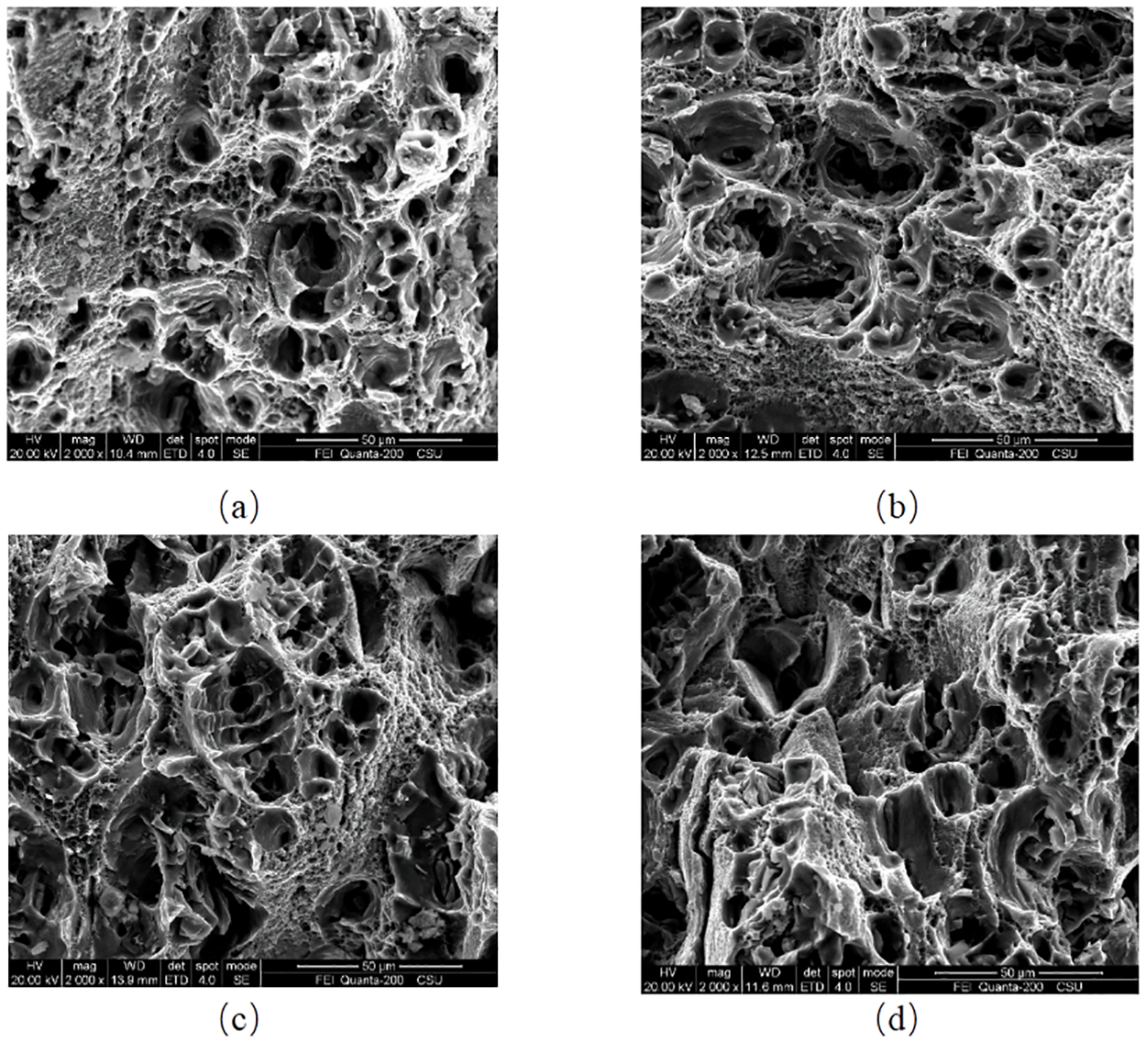
Fracture surfaces of four kind of specimens (a) smooth, (b) notched R = 90 mm, (c) notched R = 40 mm, and (d) notched R = 10 mm.

SEM micrographs of the fracture surfaces of smooth and notched specimens tested at quasi-static strain rates are compared in Fig 24. It is found that dimples had various sizes and heights in the central zone of fracture surface. The reason is that in the nucleation stage, voids grow rapidly due to high stress triaxiality. High hydrostatic pressure likely results in large dimples which may accelerate the dimple fracture. And low hydrostatic pressure leads to shear band localization and further causes shear fracture.

## Conclusions

In this paper, a series of tests were carried out to investigate the mechanical properties of AA6082-T6 under various strain rates and different stress states. Numerical simulations based on FEA were successfully performed. Primary conclusions can be drawn as follows:

- From a general view, strain rate has little influence on flow stress. However, clear rate sensitivity was observed in the strain rate range of 0.001 s^-1^ to 1 s^-1^. The softening effect of adiabatic heating counteracts the positive strain rate sensitivity at high strain rates. A Johnson-Cook constitutive model was thus proposed.
- A calibration methodology for the J-C fracture model was proposed by using the average stress triaxiality and the equivalent plastic fracture strain of the critical elements (in the central zone of the minimum cross section).
- Numerical simulations based on FEA were performed using ABAQUS. By simulating the tensile process, it was proven that the stress triaxiality is highest along the fracture cross section in the central zone, where failure begins first. The calibrated J-C fracture model was proven to be valid in predicting the fracture strain of AA6082-T6 alloy.
- Ductile fracture is the main fracture form in tensile test for the present material. Dimple fracture appears in the center of the minimum cross section due to the high level of stress triaxiality and the remaining cross sectional area is sheared to fracture. This may be caused by the rapid growth of voids in the nucleated stage due to a high stress triaxiality.
